# Visible Light Induced DLP‐Printed Oxygen‐Releasing TPMS Scaffolds Mitigate Early Hypoxia in Bone Defects

**DOI:** 10.1002/adhm.202502735

**Published:** 2025-09-23

**Authors:** Anastasia B. Timoshenko, Ali Ghasemkhani, Chanul Kim, Domenic J. Cordova, Maria Astudillo Potes, Valeria Aceves, Indranath Mitra, Justin E. Bird, Ryan S. Gray, Stephanie K. Seidlits, Benjamin D. Elder, Maryam Tilton

**Affiliations:** ^1^ Walker Department of Mechanical Engineering The University of Texas at Austin Austin TX 78712 USA; ^2^ Department of Biomedical Engineering The University of Texas at Austin Austin TX 78712 USA; ^3^ Department of Orthopedic Surgery Mayo Clinic Rochester MN 55905 USA; ^4^ College of Natural Sciences The University of Texas at Austin Austin TX 78712 USA; ^5^ Department of Applied Science William & Mary Williamsburg VA 23187 USA; ^6^ Department of Orthopedic Oncology The University of Texas MD Anderson Cancer Center Houston TX 77030 USA; ^7^ Department of Nutritional Sciences Dell Pediatric Research Institute The University of Texas at Austin Austin TX 78712 USA; ^8^ Department of Orthopedic Surgery Department of Neurologic Surgery Mayo Clinic Rochester MN 55905 USA

**Keywords:** bone tissue engineering, digital light processing, oxygen‐generating scaffolds, triply periodic minimal surface

## Abstract

Oxygen deprivation within large or poorly vascularized bone defects remains a key barrier to successful regeneration, especially during the early postimplantation period before vascular ingrowth. Here, the development of COSnPPOD (CaO_2_–Silica NP Platform for Osteogenic Development) is reported, a visible light digital light processing‐printed hydrogel scaffold that integrates oxygen‐releasing nanoparticles (NPs) within a Primitive‐type triply periodic minimal surface architecture. The scaffold combines a gelatin methacrylate‐poly(ethylene glycol) diacrylate matrix with calcium peroxide (CaO_2_)‐loaded hollow silica NPs, enabling localized, short‐term oxygen release while preserving structural fidelity. COSnPPOD scaffolds demonstrate favorable degradation kinetics, tunable stiffness, and increased protein adsorption in vitro. In a preosteoblast model, COSnPPOD maintains cell viability and supports osteogenic gene expression without cytotoxic effects. While overall gene expression is comparable to controls, a 16‐fold increased expression of phosphoprotein 1 (Spp1) suggests scaffold‐driven activation of matrix remodeling pathways. In vivo, COSnPPOD scaffolds enhance bone regeneration in a murine calvarial defect model, with significantly greater bone formation and collagen deposition than untreated defects and hydrogel controls. Additionally, vascular endothelial growth factor immunostaining is increased within the defect, consistent with a proangiogenic response, and no systemic toxicity is observed. These findings establish COSnPPOD as a promising scaffold system that combines sustained oxygenation with biomimetic geometry to support localized bone regeneration.

## Graphical abstract

Visible light‐induced digital light processing 3D printed Primitive‐triply periodic minimal surface hydrogels embed CaO_2_–Si core–shell nanoparticles to deliver short‐term oxygen during the avascular window. The scaffolds maintain cytocompatibility, elevate osteopontin in vitro, and enhance calvarial defect repair in vivo without toxicity. Coupling controlled oxygenation with curvature‐driven porosity is an advanced translatable strategy for localized early bone regeneration.

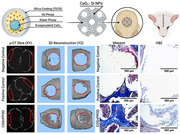

## 1 Introduction

Large bone defects suffer from poor spontaneous healing partly due to insufficient oxygen supply in the regenerating tissue.^[^ ^1^, ^2^ ^]^ Postimplantation of defect bridging constructs, the establishment of new vascular networks can be delayed, leading to hypoxic conditions within the scaffold's core.^[^ ^3^ ^]^ Severe hypoxia (<1% O_2_) disrupts cell survival, osteogenic differentiation, and tissue remodeling, ultimately compromising healing outcomes.^[^ ^4^ ^]^ Therefore, engineering strategies that proactively address early hypoxic conditions are essential for enhancing bone repair.^[^ ^5^ ^]^ Ensuring an adequate oxygen tension throughout the defect site in the early postimplantation phase is therefore crucial for cell survival, osteogenic activity, and ultimately successful bone regeneration.^[^ ^6^ ^]^ Oxygen influences multiple aspects of bone repair, from stem cell metabolism and differentiation to angiogenesis, and biomaterials that can supply oxygen have been shown to enhance tissue regeneration and reduce hypoxic damage.^[^ ^6^, ^7^ ^]^ In vitro and in vivo studies confirm that bridging the oxygen gap before vascularization can significantly improve cell proliferation and function in engineered tissues.^[^ ^5^, ^7^ ^]^ However, delivering oxygen in a controlled, sustained manner remains a key engineering challenge, as unregulated, burst release of oxygen can lead to oxidative stress (via peroxide byproducts) and tissue damage.^[^ ^7^ ^]^

In response, a variety of oxygen‐releasing biomaterials have emerged. Strategies include peroxide‐loaded hydrogels,^[^ ^8^, ^9^, ^10^ ^]^ microspheres,^[^ ^11^, ^12^, ^13^, ^14^ ^]^ and colloidal composites^[^ ^15^, ^16^, ^17^, ^18^, ^19^ ^]^ that generate oxygen on‐demand within the implant. Calcium peroxide (CaO_2_) in particular is widely used as an oxygen source due to its high oxygen yield under aqueous conditions and its relatively low cytotoxicity compared to other peroxides.^[^ ^7^, ^11^, ^20^, ^21^ ^]^ Upon hydrolysis, CaO_2_ produces hydrogen peroxide (H_2_O_2_), which subsequently decomposes into oxygen, a reaction that can boost local O_2_ levels but must be carefully controlled.^[^ ^7^, ^11^, ^21^, ^22^ ^]^ Early designs simply mixed CaO_2_ into scaffolds, which indeed elevated oxygen levels but often released oxygen too rapidly, exposing cells to bursts of H_2_O_2_ and reactive oxygen species (ROS).^[^ ^9^, ^11^ ^]^ To mitigate this, recent approaches encapsulate the peroxide in carriers or coatings that slow its exposure to water, thereby moderating the oxygen generation rate.^[^ ^11^, ^12^, ^14^, ^20^, ^22^ ^]^ For example, a previous study embedded CaO_2_ in a hydrophobic polycaprolactone (PCL) matrix to create oxygen‐releasing scaffolds with predictable release kinetics over 5 weeks.^[^ ^5^ ^]^ These scaffolds maintained physiological oxygen levels (5%–29% O_2_ depending on loading) and significantly improved cell survival and function under hypoxic conditions. Researchers have also developed an injectable hydrogel containing CaO_2_‐based microspheres that provided a steady oxygen release for up to 21 days, which in turn supported the viability and osteogenic activity of bone marrow cells even in anoxic environments.^[^ ^11^ ^]^ Such oxygen‐delivering hydrogels have demonstrated the potential to foster vascularized bone repair, especially when combined with proangiogenic or osteoinductive cues. These advances underscore that oxygen‐releasing biomaterials can accelerate bone regeneration but also highlight the need for improved control over oxygen delivery duration and dosage; the ideal parameters for oxygen delivery are the subject of current ongoing research.^[^ ^9^, ^23^, ^24^, ^25^ ^]^ Achieving a balance between sufficient oxygenation and avoidance of oxidative stress is critical.

In the context of large bone defects, hypoxia develops rapidly after implantation due to the absence of functional vasculature within the defect core. In this early “critical window” period (days to weeks postimplantation), oxygen tension can fall below 2% O_2_, impairing osteoprogenitor cell survival,^[^ ^26^ ^]^ shifting their metabolism toward glycolysis, reducing alkaline phosphatase activity, and downregulating extracellular matrix synthesis.^[^ ^27^, ^28^, ^29^, ^30^ ^]^ Hypoxia also perturbs angiogenic–osteogenic coupling by altering vascular endothelial growth factor (VEGF) signaling, disrupting osteoblast‐endothelial crosstalk, and promoting osteoclast‐mediated resorption.^[^ ^31^, ^32^, ^33^ ^]^ Consequently, early and controlled oxygen support during this prevascular phase is essential for maintaining viable cell populations, sustaining osteogenic differentiation, and preventing fibrotic tissue infiltration.

While progress has been made, relatively few studies have integrated sophisticated scaffold architectures with oxygen‐delivery functionality. Many prior systems focused on injectable formulations or simple scaffold geometries, which may not provide the optimal 3D structure for cell infiltration and load bearing in a bone defect.^[^ ^34^, ^35^, ^36^, ^37^ ^]^ Notably, recent work has begun combining oxygen release with 3D‐printed scaffolds, demonstrating the promise of merging these design aspects.^[^ ^8^, ^13^, ^19^ ^]^ For instance, a CaO_2_‐loaded PCL scaffold showed enhanced bone healing in calvarial defects, and an oxygenating hydrogel lattice prevented necrosis in a bone‐regeneration model.^[^ ^20^ ^]^ However, the influence of scaffold architecture, beyond basic porosity, on oxygen distribution and cell behavior is still an emerging area of research. We hypothesized that an optimal oxygenating scaffold should not only supply O_2_ but also feature an advanced geometry that facilitates deep cell migration, vascular ingrowth, and uniform oxygen diffusion throughout the construct.

Triply periodic minimal surface (TPMS)‐based scaffolds, characterized by continuous curvature and interconnected pore channels, have emerged as powerful design strategies for bone tissue engineering.^[^ ^37^, ^38^, ^39^ ^]^ Recent studies have shown that TPMS architectures can enhance mechanical efficiency,^[^ ^40^, ^41^ ^]^ promote uniform cell distribution, and induce osteogenic differentiation via curvature‐mediated cytoskeletal reorganization and nuclear deformation.^[^ ^42^, ^43^ ^]^ Our previous work demonstrated that Primitive‐type TPMS scaffolds fabricated from gelatin methacrylate‐poly(ethylene glycol) diacrylate (GelMA‐PEGDA) support robust vascular infiltration ex vivo.^[^ ^37^ ^]^

In this study, we designed COSnPPOD (CaO_2_–Silica NP Platform for Osteogenic Development) to bridge the critical gap between controlled oxygen delivery and biomimetic scaffold architecture for bone regeneration. The scaffold integrates three synergistic elements: First, COSnPPOD employs a Primitive triply periodic minimal surface (TPMS) geometry, offering a continuous, highly interconnected porous network with zero Gaussian curvature and high surface area.^[^ ^37^, ^42^, ^44^, ^45^ ^]^ Such topological features promote uniform oxygen diffusion, enhance nutrient transport, and guide cell migration and tissue infiltration, mimicking the architectural cues of trabecular bone.^[^ ^42^, ^44^, ^45^, ^46^, ^47^, ^48^ ^]^ Second, the scaffold embeds hollow silica nanoparticles (NPs) encapsulating CaO_2_, forming a distributed, nanoscale oxygen source. Silica NPS have been widely utilized in biomedical applications due to their tunable structure and high cargo‐loading capacity.^[^ ^49^, ^50^ ^]^ Each hollow silica NP in COSnPPOD encapsulates CaO_2_ within a porous silica shell,^[^ ^50^ ^]^ effectively creating a nanoscale reactor for oxygen generation. This design confines the peroxide in a protective matrix, limiting its immediate exposure to the aqueous surroundings and thereby tempering the rate of H_2_O_2_ release. Finally, the gelatin methacrylate‐poly(ethylene glycol) diacrylate (GelMA–PEGDA) hydrogel scaffold not only enables high‐resolution digital light processing (DLP) printing of the complex TPMS geometry but also provides a tissue‐mimicking environment to support cell attachment and proliferation.^[^ ^37^, ^51^, ^52^, ^53^ ^]^

In summary, the COSnPPOD platform represents a convergence of architectural optimization and therapeutic oxygen delivery in one construct. By leveraging an advanced TPMS pore geometry alongside an NP‐mediated oxygen release mechanism, this system is distinct from prior oxygenating biomaterials.^[^ ^8^, ^19^, ^37^, ^42^, ^45^ ^]^ Through this integrated design, COSnPPOD aims to create a spatiotemporally dynamic, pro‐osteogenic microenvironment that addresses both oxygen deficiency and structural demands during early bone healing.

## 2 Results and Discussion

### 2.1 Synthesis of CaO_2_–Si NPs and Hydrogel Encapsulation

**Figure 1:**
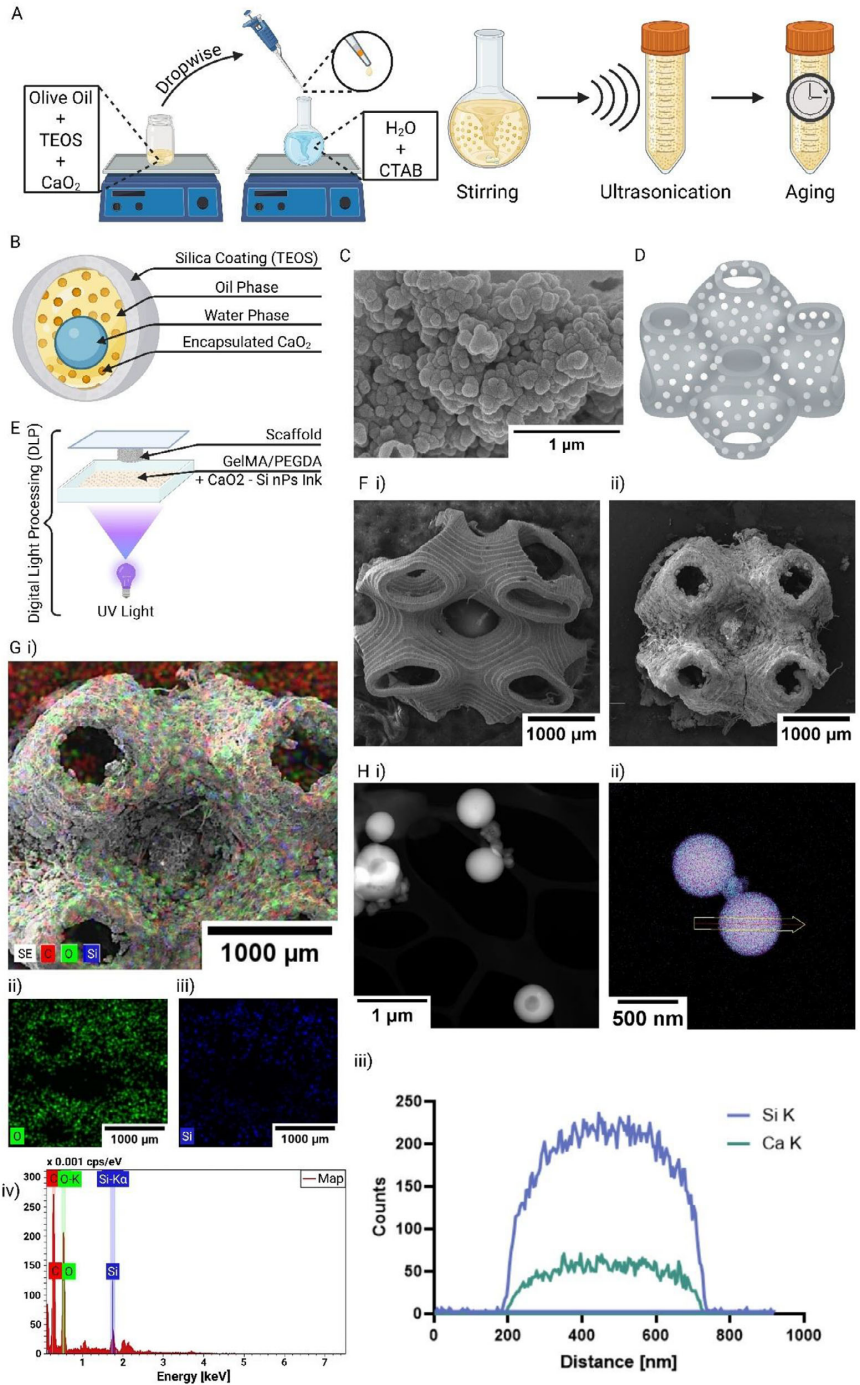
Synthesis and structural characterization of CaO_2_–Si NPs and 3D‐printed COSnPPOD scaffolds. A) Schematic illustration of the CaO_2_–Si NP synthesis using a water‐in‐oil emulsion method with CTAB as a surfactant and TEOS for silica coating. B) Schematic of the NP structure, showing a porous silica shell surrounding an aqueous core with encapsulated calcium peroxide. C) SEM image of CaO_2_–Si NPs at 50 000x magnification, showing spherical morphology with surface roughness. D) 3D model of the Primitive‐type TPMS scaffold design used for COSnPPOD. E) Schematic of the VL‐DLP 3D printing setup using a GelMA‐PEGDA ink loaded with CaO_2_–Si NPs. F) SEM images of printed scaffolds at low magnification: i) GelMA‐PEGDA control scaffold (150x), ii) COSnPPOD scaffold showing surface texture changes and visible particle loading (100x). The apparent deformation in the control scaffold reflects lyophilization‐induced collapse associated with vacuum drying and sample orientation prior to SEM, and does not indicate in situ loss of structural integrity. G) EDX analysis of COSnPPOD scaffolds: i) composite EDX elemental map (Si, O, Ca), ii, iii) individual elemental maps for oxygen and silicon, and iv) EDX spectrum confirming elemental composition. H) STEM images of core–shell NPs at magnifications of i) 30 000x and ii) 100 000x, highlighting the spherical morphology and core–shell structure, and the accompanying iii) EDX elemental mapping demonstrating the spatial distribution of silicon (Si) and calcium (Ca) within the NPs.

To establish controlled and sustained oxygen delivery within the COSnPPOD scaffold, we synthesized CaO_2_‐loaded hollow silica NPs using an oil‐in‐water nanoemulsion method (**Figure** **1**A), resulting in a hollow silica nanosphere with a layered water‐in‐oil core and CaO_2_ stored within the oil phase of the NP core, protected by the silica shell until the time of release (Figure 1B).^[^ ^50^ ^]^ The hollow silica shell served as a protective barrier to modulate CaO_2_ hydrolysis, mitigating burst release and allowing for sustained oxygen generation over time. Scanning electron microscopy (SEM) imaging of the resulting NPs confirmed their spherical shape and their nanoscale dimensions (Figure 1C). The synthesized NPs were incorporated into a GelMA‐PEGDA precursor (i.e., photocrosslinkable ink)^[^ ^37^ ^]^ through direct mixing, which was then used with our visible light‐induced digital light processing (VL‐DLP) (bio)printer (LUMEN X, Gen3, CELLINK) to 3D print Primitive‐based TPMS scaffolds (Figure 1D,E).^[^ ^37^ ^]^ The resulting scaffold comprised the proposed COSnPPOD treatment system. Positive control GelMA‐PEGDA scaffolds were printed without CaO_2_‐loaded hollow silica NPs.

SEM imaging (Figure 1F) confirmed that the VL‐DLP scaffolds faithfully reproduced the designed Primitive TPMS geometry with interconnected pore networks. Compared to control GelMA‐PEGDA scaffolds (Figure 1F,i), COSnPPOD scaffolds (Figure 1F,ii) exhibited distinct microstructural surface features, including fine filamentous or fibrillar structures not observed in control scaffolds. These features may result from NP‐induced changes during the DLP crosslinking process, potentially involving localized scattering effects^[^ ^54^ ^]^ or microphase separation.^[^ ^55^ ^]^ Energy‐dispersive X‐ray spectroscopy (EDX) elemental mapping across scaffold cross‐sections detected Si/O/Ca signals throughout the COSnPPOD walls, indicating spatially uniform NP distribution at the scaffold scale (Figure 1G). Scanning transmission electron microscopy (STEM) imaging resolved the core–shell structure at high magnification (30 000x and 100 000x), and STEM‐EDX maps showed Si enriched in the shell and Ca concentrated in the core, consistent with the intended structure (Figure 1H). Additionally, mass swelling and gel fraction assessments were performed on the 3D printed scaffolds (Figure S1A,B, Supporting Information). Fully quantitative 3D mapping of submicrometer CaO_2_–Si nanoparticles in millimeter‐scale, hydrated hydrogels remains challenging. Fluorescent labeling of silica (e.g., aminosilane‐dye conjugates) alters surface chemistry, which can change aggregation, protein adsorption, and release, while confocal microscopy in thick gels is depth‐limited and biased. Future work should consider regional digests with inductively coupled plasma optical emission spectroscopy (ICP‐OES)/inductively coupled plasma mass spectroscopy (ICP‐MS) or cryo‐section STEM‐EDX line profiling to obtain region‐wise NP distribution quantification without modifying the nanoparticles.

The swelling ratio of the control samples was measurably higher, with the result showing statistical significance (*p* < 0.05) (Figure S1, Supporting Information); however, based on micro‐CT analysis and histology images, these samples have lower porosity. One reason for this discrepancy is the hydrophobicity of the NPs, resulting in a lower fluid uptake into the COSnPPOD scaffolds. Additionally, the gel fraction values appeared to be similar, signifying that COSnPPOD does not have a significant negative effect on crosslinking density.

**Figure 2:**
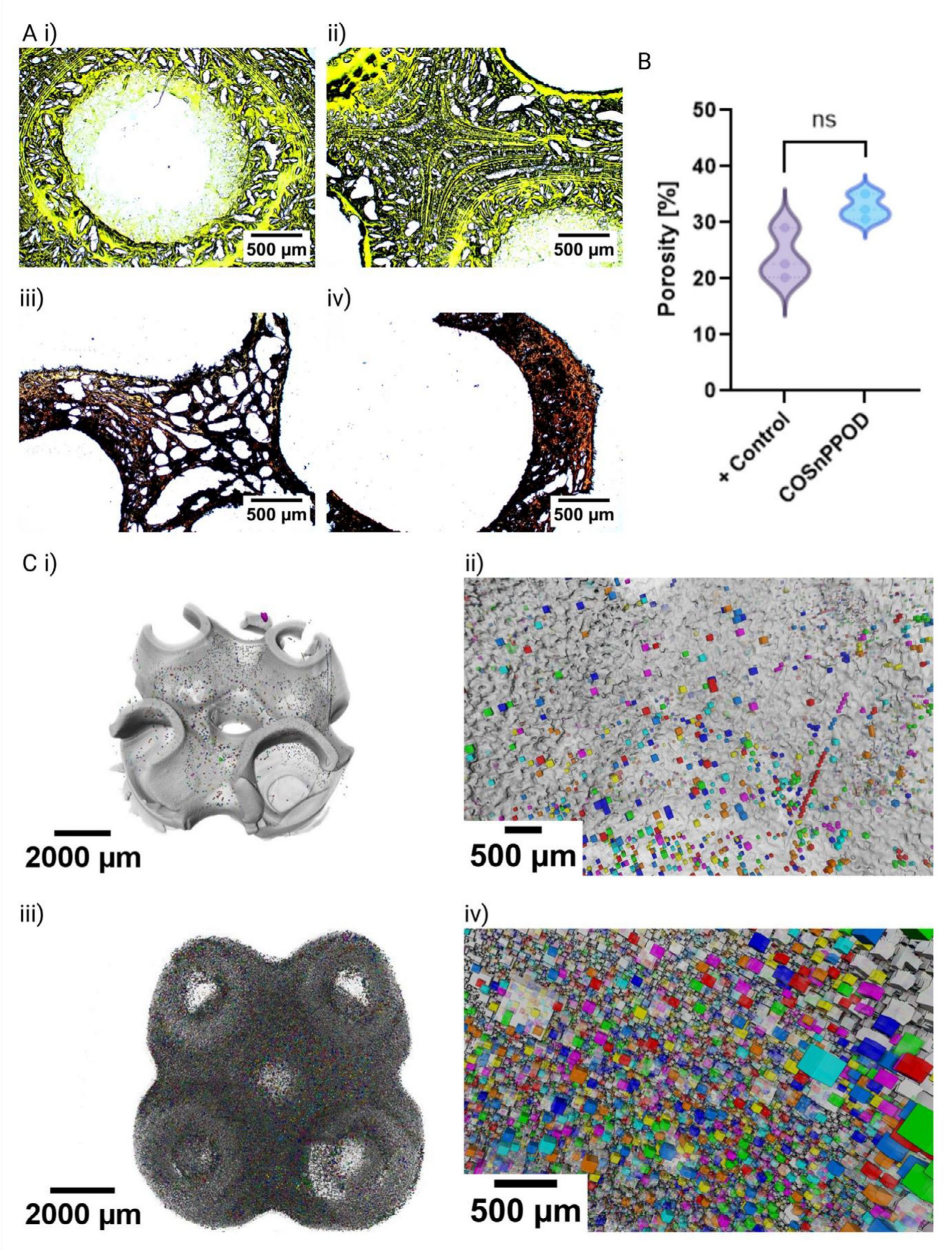
Porosity characterization of 3D‐printed COSnPPOD scaffolds. A) Representative XFAST histological staining of cryosectioned scaffolds illustrating intraconstruct microarchitecture. A, i‐ii) GelMA‐PEGDA control showing tartrazine uptake i, ii: two nonoverlapping regions). A, iii, iv) COSnPPOD scaffold exhibiting purple–burgundy staining from Alcian Blue/Safranin O uptake by CaO_2_Si NPs, along with distinct microporosity and layer resolution: iii) strut/interior region; iv) macropore wall region (nonoverlapping and sampled from a distant field relative to iii). B) %Porosity of the hydrogels as calculated from images of cryosectioned hydrogel samples using ImageJ image processing software (*n* = 3 scaffolds/group); COSnPPOD hydrogels displayed higher porosity, though the results are not statistically significant. C) Representative 3D reconstructed model of µCT data of i, ii) control and iii, iv) COSnPPOD scaffolds (*n* = 3 scaffolds/group). Cross‐sectional view demonstrating a higher apparent porosity in COSnPPOD compared to positive control. Quantitative results are presented as mean ± SD. Statistical significance relative to positive control was calculated using Welch's *t*‐test; *p* < 0.05 is designated as statistically significant.

Histological analysis using a fluorescent antibody staining technique (XFAST)^[^ ^56^ ^]^ on cryosectioned scaffold slices further revealed the internal microstructure of the constructs (**Figure** **2**A). In control scaffolds, primarily tartrazine staining was observed, consistent with the ink formulation. In contrast, COSnPPOD scaffolds exhibited additional Alcian Blue and Safranin‐O uptake by the CaO_2_–Si NPs, appearing as burgundy–purple inclusions distributed within the scaffold matrix, confirming their homogeneous distribution. Notably, the COSnPPOD scaffolds also exhibited increased microporosity compared to controls, with the presence of larger, irregular pores within the hydrogel matrix. These nondesigned micropores likely arose from NP‐induced light scattering^[^ ^54^, ^57^, ^58^, ^59^ ^]^ or local crosslinking variations during the DLP fabrication process. Despite these microstructural differences, the overall layer‐by‐layer architecture and TPMS‐derived macrogeometry were preserved in the COSnPPOD scaffolds. We quantified section‐level porosity using a single, fixed ImageJ pipeline (2D cross‐sectional void fraction). COSnPPOD sections showed a higher mean 2D porosity than controls, although the difference was not statistically significant (Figure 2B).

We next assessed the 3D pore architecture with phosphotungstic acid (PTA)‐enhanced micro‐computed tomography (µCT) of hydrated scaffolds at 5 µm isotropic voxels. Reconstructions confirmed faithful reproduction of the Primitive TPMS macrogeometry in both groups (Figure 2C). Quantitatively, the image‐scale total porosity (porosity detectable at 5 µm voxels) averaged 21.5% in controls and 26.09% in COSnPPOD (*n* = 3 per group were scanned). Although this directional increase is concordant with histology and the µCT cross‐sectional views, which appear more porous in COSnPPOD, it did not reach statistical significance at this resolution and sample size. Pore size distribution analysis (Figure S1C, Supporting Information) showed a broader distribution in COSnPPOD and a higher maximum pore radius (98 µm in control vs 110 µm in COSnPPOD). The apparent discrepancy between visibly greater porosity in COSnPPOD (histology and µCT cross‐sections) and the modest, nonsignificant difference in bulk 3D porosity is attributable to two well‐recognized technical factors that we controlled and report explicitly: i) At 5 µm voxels, pores smaller than ≈15 µm are not robustly quantifiable; boundary voxels bias tiny voids toward the solid phase, systematically under‐estimating fine microporosity and attenuating between‐group contrasts. ii) To maximize reproducibility, all reconstruction and thresholding parameters were held constant across samples. This conservative choice reduces operator bias but can compress small group differences when grayscale contrast (e.g., PTA uptake) varies subtly.

### 2.2 NP Inclusion Enables Gradual Oxygen Release and Modulates Print Physics and Scaffold Biophysical Properties

**Figure 3:**
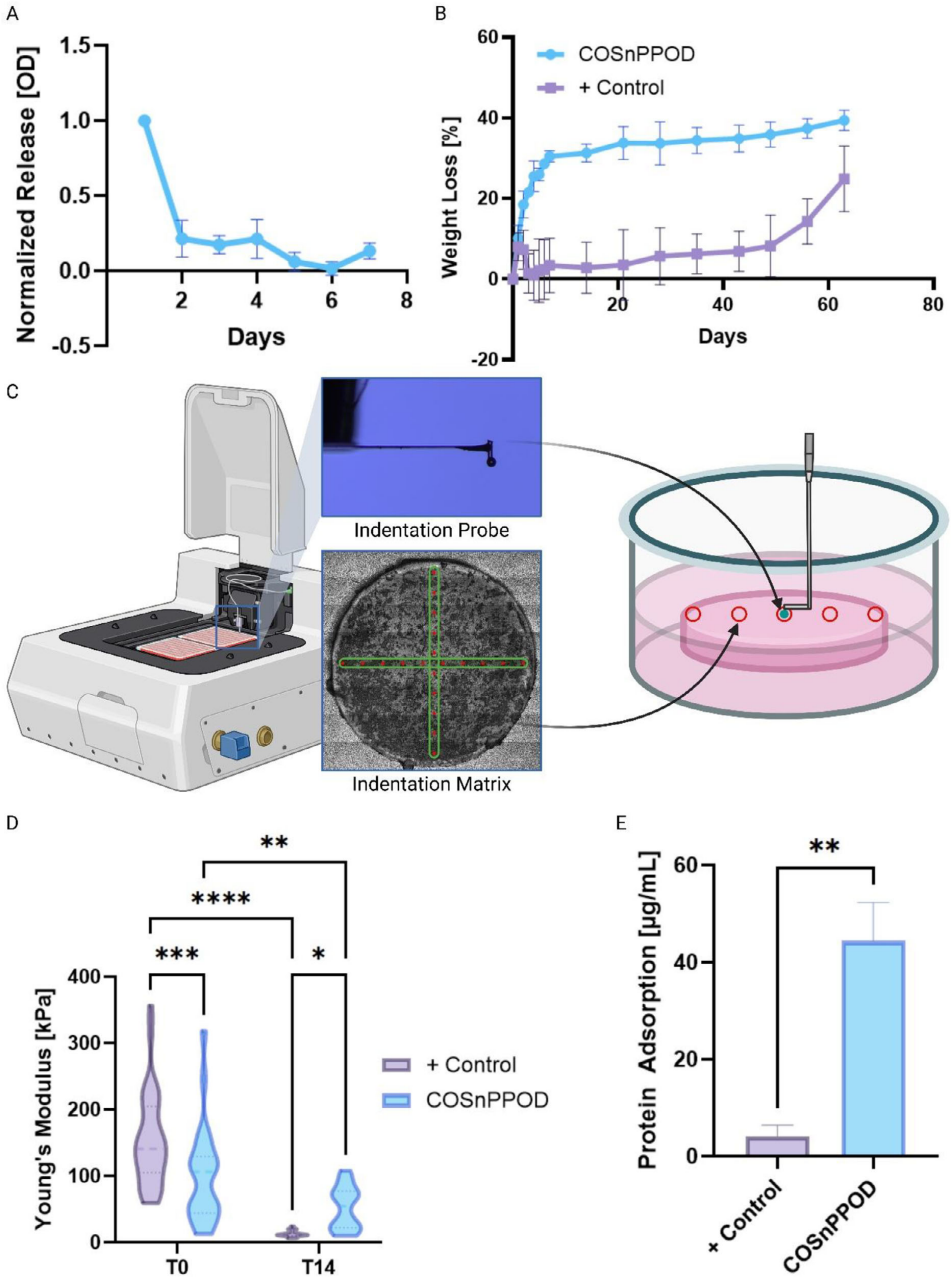
In vitro characterization of oxygen release, biodegradation, mechanical properties, and protein adsorption of COSnPPOD scaffolds. A) Relative oxygen release from COSnPPOD scaffolds over 7 days, normalized to the initial levels: OD release value at day 1. Peak release occurred within the first 24 h, followed by gradual tapering. B) Biodegradation profiles of porous control and treatment scaffolds measured by percentage weight loss over 8 weeks. COSnPPOD scaffolds exhibited a distinct two‐phase degradation pattern with faster initial mass loss. C) Schematic of nanoindentation setup used to assess local hydrogel stiffness. Micromechanical profiling was done at baseline (T0) and day 14 (T14). Nanoindentation was performed on flat disk specimens (same VL‐DLP process and NP loading) for contact stability; the paired disk mass‐loss profiles are shown in Figure S2A (Supporting Information). D) Quantification of Young's modulus using optical fiber interferometry nanoindentation. COSnPPOD scaffolds displayed lower initial stiffness but retained mechanical properties better than controls on day 14. E) Protein adsorption from culture medium measured by MicroBCA assay. COSnPPOD scaffolds showed significantly greater protein binding than controls, likely due to surface charge and increased microporosity. A minimum of *n* = 3 scaffolds per study group were used for all experiments and biological replicates. Quantitative results are presented as mean ± SD. Statistical significance relative to positive control was calculated using two‐way ANOVA and Fisher's LSD test for multiple comparisons D) and Welch's *t*‐test E); *p* < 0.05 is designated as statistically significant. Statistical significance: *: *p* < 0.05, **: *p* < 0.01, ***: *p* < 0.001, ****: *p* < 0.0001.

We quantified the temporal pattern of oxygen‐generating NP release from COSnPPOD over 7 days as a normalized profile to emphasize kinetics rather than absolute amounts (**Figure** **3**A). Release peaked within 24 h and declined thereafter; by day 7 the daily signal was about 87% lower than day 1, consistent with the early‐stage therapeutic window that aligns with the avascular phase of bone repair.^[^ ^60^, ^61^ ^]^

To assess how NP loading affected degradation and mechanics, we paired enzymatic mass‐loss assays^[^ ^37^ ^]^ with nanoindentation. Porous COSnPPOD scaffolds exhibited faster initial mass loss than NP‐free controls (Figure 3B; disk biodegradation profiles presented in Figure S2A, Supporting Information).^[^ ^37^ ^]^ This enhanced degradation is likely due to a combination of higher microporosity and early oxygen release, which together increase fluid penetration and matrix erosion.^[^ ^62^ ^]^ In contrast, the control scaffolds degraded more gradually, consistent with a slower enzymatic breakdown of a more intact hydrogel matrix. Incorporating silica‐based CaO_2_ particles into the GelMA‐PEGDA ink unavoidably influences the VL‐DLP printing physics. The opaque NP phase scatters incident visible light, effectively broadening the polymerization zone and reducing the effective cure depth.^[^ ^54^, ^57^, ^58^, ^59^ ^]^​ As demonstrated in Figure 2, in our VL‐DLP process, the resulting scattered light gave rise to the fine filamentous features and “nondesigned” micropores observed in COSnPPOD scaffolds. Such microstructural irregularities stem from localized under‐polymerization as light intensity drops off within the particle‐laden ink. Notably, the overall TPMS geometry was still preserved, indicating that our NP loading remained within a printable regime, and did not inhibit the photopolymerization process. While high NP content can challenge print fidelity, the controlled introduction of CaO_2_–Si NPs in COSnPPOD scaffolds was achieved without macroscale geometric loss, at the cost of a modest increase in microscale porosity. Importantly, such microporosity may be serendipitously beneficial for tissue engineering, as scaffold porosity is known to promote cell attachment, proliferation, and osteogenic differentiation​ by increasing surface area for cell–material interaction.^[^ ^48^, ^63^, ^64^, ^65^ ^]^

Mechanical testing using an optical fiber‐based interferometry nanoindenter on disk‐shape scaffolds (Figure 3C,D) showed that COSnPPOD scaffolds had slightly lower Young's modulus values on Day 0 (T0) (Effective Young's Modulus results in Figure S2B, Supporting Information), consistent with reduced crosslinking efficiency due to light scattering by the opaque NP‐laden ink. However, over time, the residual inorganic phase provided a reinforcing effect, resulting in higher stiffness in COSnPPOD scaffolds after 14 days of enzymatic degradation. This behavior reflects composite enrichment and the local measurement domain of nanoindentation. As the matrix erodes, rigid CaO_2_–Si nanoparticles remaining in the walls increase their effective volume fraction within the load‐bearing skeleton, elevating the solid‐phase modulus despite greater overall mass loss. During the nanoindentation, the contact increasingly coincides with NP‐enriched and protein‐adsorbed regions, further elevating the measured local modulus by day 14. Thus, bulk mass‐loss and local modulus trends do not covary. Furthermore, the reactive nature of CaO_2_ NPs contributes to the degradation profile of the scaffolds.^[^ ^66^ ^]^ The release of oxygen and byproducts from COSnPPOD creates additional microporosity, accelerating water infiltration in the early stages. This behavior aligns with the observed rapid mass loss in COSnPPOD scaffolds relative to controls. Finally, a protein adsorption assay (Figure 3E) revealed that COSnPPOD scaffolds adsorbed significantly more total protein from culture medium compared to controls. While gelatin‐based hydrogels are inherently protein‐adhesive, this enhancement is attributed to the greater surface area and the presence of charged silicate interfaces from the embedded NPs, which can sequester bioactive proteins and facilitate extracellular matrix deposition.^[^ ^67^, ^68^ ^]^

Since peroxide chemistry can shift medium pH,^[^ ^33^ ^]^ and extracellular pH tightly regulates osteoblast proliferation/differentiation and osteoclast activity,^[^ ^33^ ^]^ we ran a cell‐free study in α‐MEM (no media changes) to track pH transients. Here, we observed a single early drop on day 3 followed by stabilization (Figure S2C, Supporting Information). This pattern is consistent with hydrolysis of ester linkages in the PEGDA network which generates carboxylic acids rather than persistent alkalinization from CaO_2_ (which in many systems elevates pH to about 8–9).^[^ ^69^ ^]^ Thus, in our mixed GelMA‐PEGDA backbone formulation, the net early acidification likely reflects PEGDA‐ester hydrolysis dominating over CaO_2_’s basic contribution in the closed, bicarbonate‐buffered system.^[^ ^69^ ^]^ In this study, absolute dissolved O_2_ values were not measured because point probes can perturb local O_2_, exhibit boundary‐layer artifacts and are highly sensitive to boundary and convective conditions, factors that can bias quantitation in porous scaffolds.^[^ ^70^, ^71^ ^]^ Planar or optical approaches mitigate some issues but still report local rather than construct‐wide O_2_ and are sensitive to dye calibration and photophysics.^[^ ^70^, ^72^ ^]^ Fluorescent dye labeling for release quantification, such as using RITC,^[^ ^73^ ^]^ Fluorescein,^[^ ^74^, ^75^ ^]^ FITC,^[^ ^74^, ^75^ ^]^ WA6,^[^ ^75^ ^]^ is a commonly used alternative method. However, the addition of dyes to the NP composition via methods, such as covalent attachment or physical entrapment may affect the chemical and physical properties of the NPs, thus modifying the resulting release kinetics. Furthermore, dye leaching over time can also affect the release measurements.^[^ ^74^ ^]^ Therefore, we prioritized a label‐free kinetic readout here. Although normalized NP‐release traces capture therapeutically relevant timing, we note that they do not report absolute O_2_ flux or H_2_O_2_. Future studies will combine i) in‐scaffold planar O_2_ sensors for spatiotemporal mapping, ii) microdialysis‐coupled assays for H_2_O_2_, and iii) pH‐stable buffers to deconvolve hydrogel hydrolysis from peroxide chemistry.^[^ ^33^, ^48^ ^]^

### 2.3 In Vitro Cytocompatibility, Proliferation, and Osteogenic Gene Response to COSnPPOD Scaffolds

**Figure 4:**
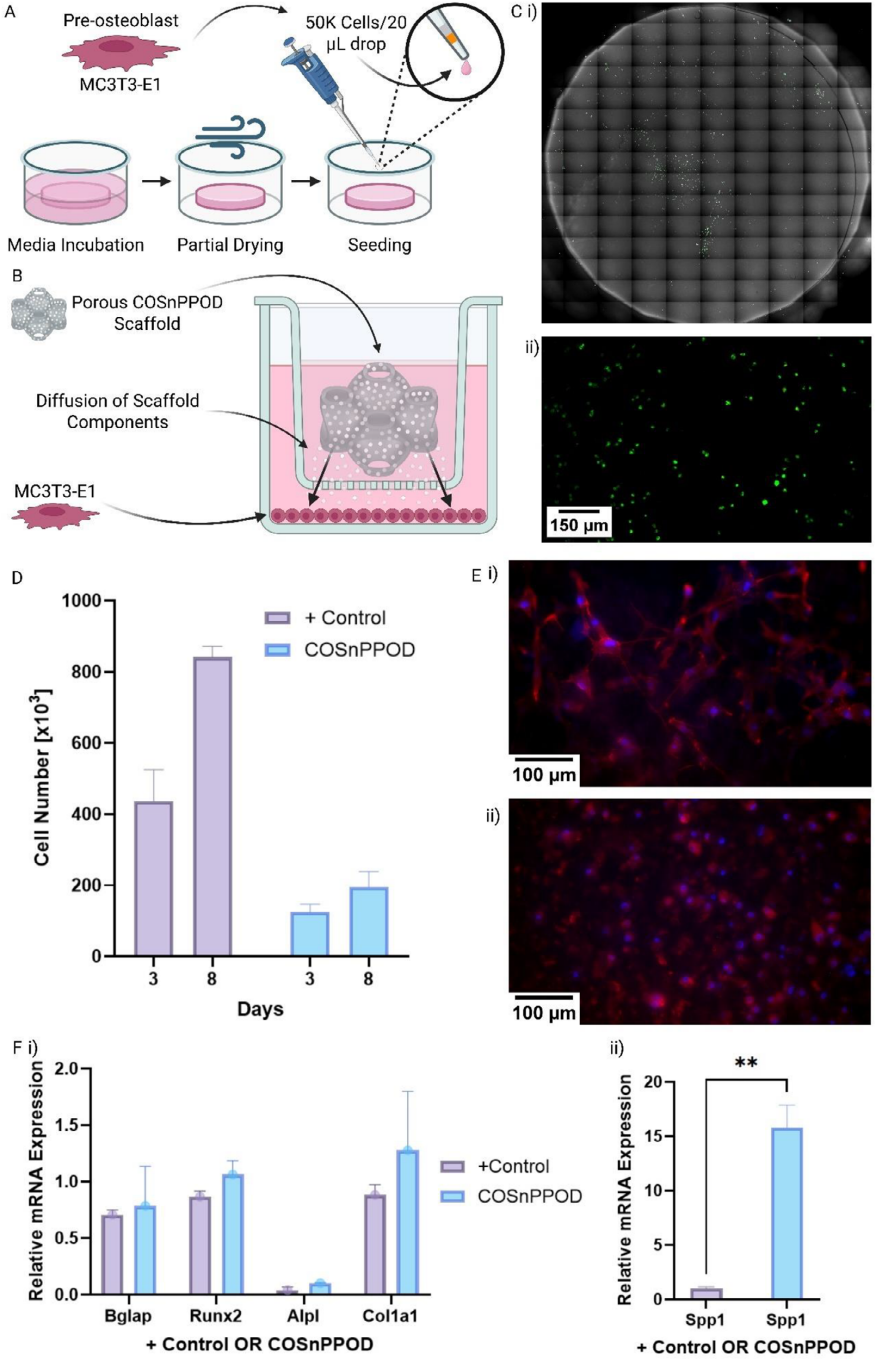
In vitro evaluation of preosteoblast viability and osteogenic gene expression in response to COSnPPOD scaffolds. A,B) Schematic of the experimental setup. MC3T3‐E1 preosteoblasts were either directly seeded on scaffolds for imaging‐based cytocompatibility assays or cultured in a transwell system with scaffolds to assess paracrine effects of oxygen release on osteogenic gene expression. C) Live/Dead fluorescence staining on day 3 postseeding (i.e., day 0 of osteogenic induction): i) stitched tile‐scan of a control scaffold and ii) representative magnified view (10x). COSnPPOD scaffolds were not imaged due to strong autofluorescence and dye uptake by embedded CaO_2_–Si NPs. D) Cell proliferation quantified via CyQUANT assay on days 3 and 8, normalized for sample dilution. COSnPPOD exposure resulted in reduced proliferation relative to controls. E) Immunofluorescence staining of F‐actin (red) and nuclei (blue) at 48 h showing well‐spread morphology in both i) control and ii) COSnPPOD conditions. F) RT‐qPCR analysis of osteogenic gene expression on day 8: i) canonical osteogenic markers (*Runx2, Alpl, Col1a1, Bglap*) exhibited comparable or modestly increased expression in COSnPPOD cultures; ii) *Spp1* (osteopontin) was significantly upregulated in the COSnPPOD group (*p* < 0.01), suggesting early pro‐remodeling activation. All experiments were performed using *n* ≥ 3 biological replicates per group. Quantitative results are presented as mean ± SD. Statistical significance relative to positive control was calculated using Welch's *t*‐test; *p* < 0.05 is designated as statistically significant. Statistical significance: *: *p* < 0.05, **: *p* < 0.01, ***: *p* < 0.001, ****: *p* < 0.0001.

To evaluate the biocompatibility and osteogenic potential of the COSnPPOD scaffold, two complementary in vitro models were employed: 1) direct cell seeding on disk‐shaped scaffolds for initial cytocompatibility assays and imaging‐based assessment of cell morphology and cell–matrix interactions (**Figure** **4**A), and 2) a transwell coculture system to examine paracrine effects of the TPMS porous scaffold on MC3T3‐E1 preosteoblasts (Figure 4B).

Cytocompatibility was assessed using both Live/Dead staining and CyQuant proliferation assay (Figure 4C,D), confirming desired cell viability on both control and COSnPPOD scaffolds. Particularly, CyQUANT analysis showed reduced proliferation in COSnPPOD‐exposed cultures at days 3 and 8 relative to controls (Figure 4D; fluorescence corrected for sample dilution), in the absence of overt cytotoxic morphology. Given the controlled, short‐term oxygenation of COSnPPOD, a parsimonious interpretation is transient cytostasis, likely reflecting early‐phase redox/pH transients that temper cell cycling while preserving viability, rather than toxicity.^[^ ^5^, ^23^, ^76^ ^]^ Importantly, such a proliferation‐to‐differentiation trade‐off is a hallmark of osteoblast maturation and aligns with the morphology assessments.^[^ ^77^, ^78^, ^79^ ^]^ Specifically, at 48 h, F‐actin/nuclei staining revealed distinct morphologies between groups (Figure 4E): control cultures exhibited elongated cells with prominent filopodia‐like protrusions forming intercellular bridges, whereas COSnPPOD‐exposed cells displayed broader lamellipodial spreading with fewer long protrusions at the early time point. We imaged at 48 h intentionally (before rapid proliferation confounds morphology) to capture primary adhesion and spreading. This shift toward lamellipodial, adhesion‐mature phenotype in the treatment condition is consistent with: i) enhanced protein adsorption on COSnPPOD (Figure 3E), which increases effective integrin‐ligand presentation and favors isotropic spreading over filopodial searching,^[^ ^80^, ^81^ ^]^ and ii) the modestly lower initial micromechanical stiffness measured in COSnPPOD (Figure 3D), which is known to bias actin organization toward cortical/lamelliform spreading on softer matrices^[^ ^82^ ^]^ rather than elongated, protrusion‐rich morphologies. Together, these data indicate that the treatment scaffold supports robust early attachment with a morphology associated with adhesion maturation rather than exploratory motility.^[^ ^83^ ^]^

To assess the osteogenic potential of the COSnPPOD scaffolds, quantitative real‐time quantitative polymerase chain reaction (RT‐qPCR) was performed on MC3T3‐E1 cells cultured for 8 days under osteogenic conditions using the transwell coculture model. Expression profiles of four canonical osteogenic genes were analyzed: Runt‐related transcription factor 2 (*Runx2*), alkaline phosphatase (*Alpl*), collagen type I alpha 1 (*Col1a1*), and bone gamma‐carboxyglutamic acid‐containing protein (*Bglap*, also known as osteocalcin). *Runx2* is a master transcription factor that governs early osteoblast lineage commitment,^[^ ^84^ ^]^ while *Alpl* is a marker of matrix vesicle formation and early mineralization.^[^ ^85^, ^86^ ^]^ *Col1a1* encodes the primary structural protein in bone extracellular matrix,^[^ ^87^ ^]^ and Bglap reflects late‐stage osteoblast differentiation and mineral deposition.^[^ ^88^ ^]^

Across these four genes, no statistically significant differences in expression were observed between cells exposed to COSnPPOD scaffolds and those in the control group (Figure 4F,i). The absence of downregulation in any of these genes suggests that oxygen release from COSnPPOD does not impair the osteogenic differentiation trajectory of preosteoblasts. Mean expression levels of Runx2 and Col1a1 were modestly elevated in the COSnPPOD group, but the variation within groups limited statistical interpretation.

Interestingly, expression of secreted phosphoprotein 1 (*Spp1*, also known as osteopontin) was markedly upregulated in the COSnPPOD group, showing an ≈16‐fold increase compared to control (15.81 ± 2.06 vs 1.00 ± 0.15) (Figure 4F,ii). Osteopontin (OPN) is a multifunctional glycoprotein involved in bone remodeling, and is a key marker of osteogenic differentiation, often associated with the later stages of osteoblast maturation.^[^ ^89^ ^]^ Its significant upregulation in the COSnPPOD group suggests that the scaffold created a more favorable microenvironment for osteogenic progression. In similar studies, bioactive scaffolds that modulate oxygen and redox conditions have likewise been shown to boost OPN expression alongside other bone genes, correlating with enhanced osteogenic differentiation.^[^ ^1^, ^20^, ^89^ ^]^ Therefore, the elevated *Spp1* in COSnPPOD cultures, together with preserved expression of *Runx2, Alpl, Col1a1*, and *Bglap*, and the observed adhesion‐mature morphology, supports progression toward a remodeling‐competent osteoblastic phenotype rather than indicating cytotoxic stress. Overall, the data indicate that while COSnPPOD exposure does not significantly alter the expression of genes directly involved in osteoblast maturation, it does elicit a matrix‐responsive transcriptional response, most notably through osteopontin. This suggests the scaffold may prime the local environment for tissue remodeling and bone formation without disrupting cellular lineage progression. To further examine this hypothesis, we next tested this in a well‐established critical‐size murine calvarial defect mode.

### 2.4 In Vivo Evaluation Confirms Biocompatibility and Enhanced Bone Regeneration by COSnPPOD

**Figure 5:**
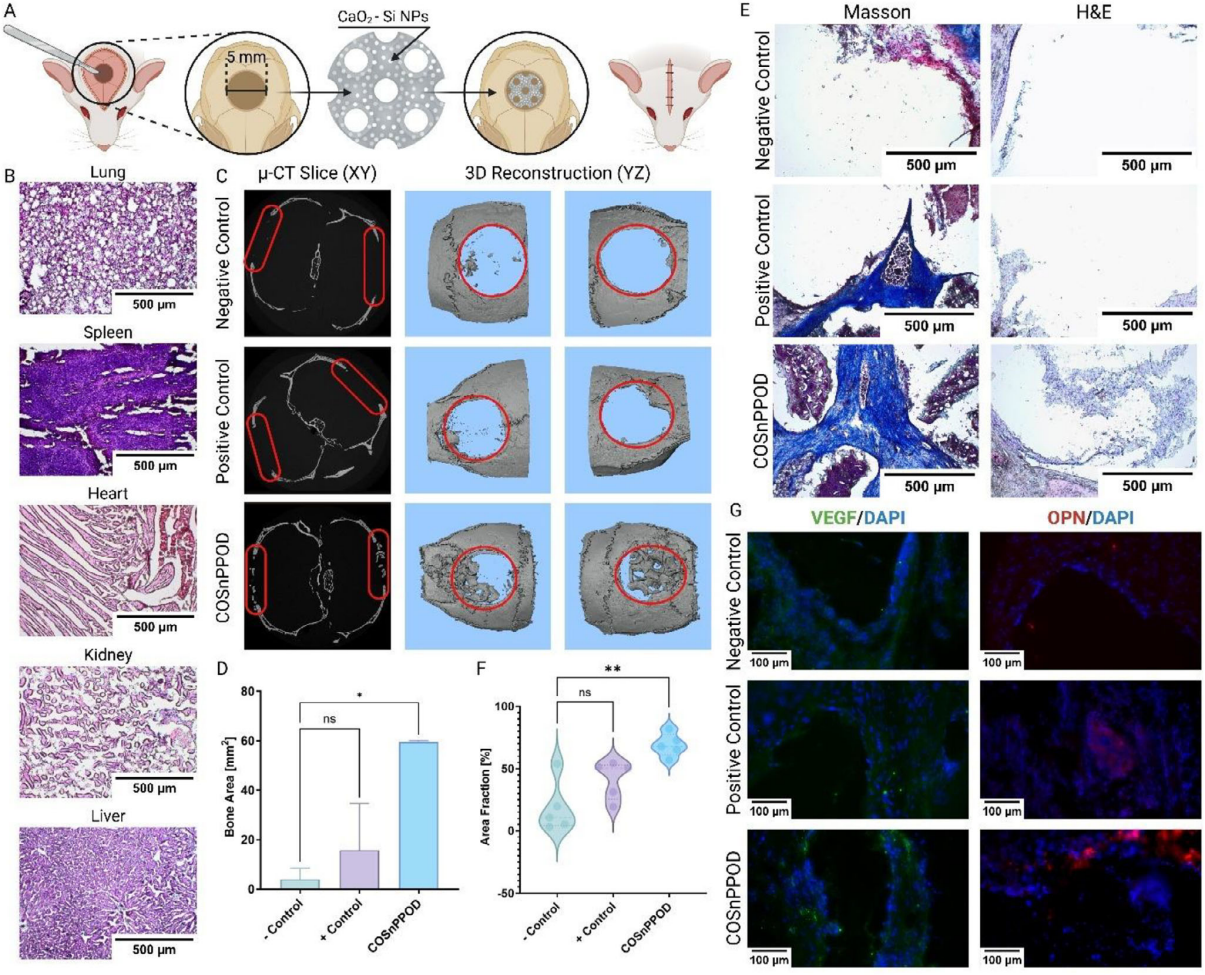
In vivo evaluation of bone regeneration and systemic biocompatibility of COSnPPOD scaffolds in a murine calvarial defect model. A) Schematic of the surgical procedure and scaffold implantation in a 5 mm critical‐sized calvarial defect. COSnPPOD scaffolds contain CaO_2_–Si NPs embedded in a 3D‐printed Primitive TPMS hydrogel structure. B) H&E staining of major off‐target organs (lung, spleen, heart, kidney, liver) harvested at 8 weeks postimplantation (*n* = 3 slices per biological replicates). No signs of inflammation, fibrosis, or tissue damage were observed, confirming scaffold biocompatibility. C) Representative µCT data showing XY plane slices (left) and 3D reconstructions in the YZ plane (middle and right) for each treatment group. Red circles indicate the region of interest (ROI). D) Quantification of bone area in ROI from µCT reconstructions. COSnPPOD scaffolds significantly increased bone regeneration compared to both negative and positive control groups (*p* < 0.05). E) Representative histological staining of calvarial tissue sections. H&E staining (top) reveals cell infiltration and defect bridging; Masson's Trichrome staining (bottom) highlights collagen matrix deposition and early bone formation (*n* = 3 slices per biological replicates). F) Quantification of collagen area fraction from Masson's Trichrome images (*n* = 5 images per group). COSnPPOD scaffolds supported significantly higher collagen deposition than both control groups (*p* < 0.01). G) Representative immunofluorescent staining for OPN (red) and VEGF (green) with DAPI (blue). Exposure and processing were kept constant across groups (*n* = 3 slices per biological replicates). Total of *n* = 4 biological replicates (i.e., mice) were used per study group. Statistical significance relative to positive control was calculated using ordinary one‐way ANOVA D) and one‐way Brown–Forsythe and Welch ANOVA F); *p* < 0.05 is designated as statistically significant. Statistical significance: *: *p* < 0.05, **: *p* < 0.01, ***: *p* < 0.001, ****: *p* < 0.0001.

To assess the therapeutic efficacy and systemic biocompatibility of COSnPPOD scaffolds, we employed a well‐established murine calvarial defect model (**Figure** **5**A). Critical‐sized defects (5 mm) were treated with either COSnPPOD scaffolds, control GelMA–PEGDA scaffolds (positive control), or left untreated (negative control). The aim was to evaluate not only the local bone regeneration potential of COSnPPOD but also the biological compatibility of its oxygen‐releasing chemistry in vivo. All animals survived to the study endpoint (8 weeks), with no visible signs of distress or adverse effects. Gross health monitoring and postmortem histological analysis of major off‐target organs, including liver, kidney, heart, and spleen, revealed no significant inflammatory infiltration or tissue damage (Figure 5B). These findings indicate that COSnPPOD scaffolds do not elicit systemic toxicity or inflammation at 8 weeks postimplantation, confirming their safety profile and supporting further translational development.

High‐resolution µCT imaging of calvarial explants demonstrated significantly enhanced bone regeneration in the COSnPPOD group compared to both control and untreated groups (Figure 5C,D). Quantitative analysis of bone surface area showed a near threefold increase over GelMA‐PEGDA scaffolds, with 3D reconstructions revealing extensive mineral bridging across the defect. Histological analyses corroborated the µCT findings. Hematoxylin and Eosin (H&E) staining revealed substantial cellular infiltration and early tissue remodeling in the COSnPPOD group (Figure 5E). Masson's Trichrome staining further demonstrated dense collagen deposition throughout the defect, indicative of organized matrix formation and osteogenesis. Quantification of collagen area fraction showed a statistically significant increase in the COSnPPOD group compared to both positive and negative controls (Figure 5F), supporting a proregenerative effect.

To probe local osteogenic remodeling and angiogenic activity within the regenerating defect, we performed immunofluorescent staining for osteopontin (OPN) and VEGF on calvarial sections harvested at 8 weeks (Figure 5G). OPN, a matricellular protein associated with active bone remodeling and mineralizing osteoid,^[^ ^90^ ^]^ was minimally detected in the negative control group and sparse in the positive control samples. In contrast, COSnPPOD explants displayed intense OPN signal localized along the defect‐scaffold interface and within newly deposited matrix spanning the defect. This pattern aligns with the increased collagen area fraction (Figure 5F) and supports an environment of ongoing osteogenesis and matrix maturation at the COSnPPOD site.

VEGF staining revealed more frequent and spatially extensive VEGF‐positive cells/microvessel‐like structures in the COSnPPOD group relative to both positive and negative controls (Figure 5G). Although the staining is presented qualitatively, the distribution and density of VEGF signal are consistent with heightened neovascularization within the regenerating tissue.^[^ ^91^, ^92^ ^]^ Together with the µCT and histology, these findings suggest that short‐term oxygenation combined with TPMS architecture supports a viable, proangiogenic microenvironment that sustains osteogenic–angiogenic coupling during defect repair. We note that OPN can be produced by multiple cell types involved in bone repair (e.g., osteoblasts, osteoclasts, macrophages) and therefore reports a remodeling state rather than a single lineage. Likewise, VEGF immunoreactivity indicates proangiogenic activity but not vessel maturity.^[^ ^91^ ^]^ Future studies should consider incorporating endothelial/pericyte markers and perfusion imaging to assess vascular density and maturation.

These outcomes are attributed to the dual functionality of the COSnPPOD scaffold. The encapsulated CaO_2_–Si NPs provide short‐term oxygen release, potentially mitigating hypoxia and supporting cellular metabolism during the early avascular phase of healing. In parallel, the scaffold's architecturally optimized Primitive‐type TPMS geometry offers continuous curvature and interconnected porosity, facilitating cell migration, vascular infiltration, and spatially uniform tissue deposition. Previous studies have shown that TPMS‐based scaffolds, particularly those with Primitive curvature, promote early osteoconduction and neotissue homogeneity more effectively than stochastic or lattice‐based structures.^[^ ^38^, ^39^, ^42^ ^]^ Additionally, in our previous published work,^[^ ^37^ ^]^ GelMA‐PEGDA 10 w/w% formulation, when combined with a TPMS Primitive scaffold architecture, elicited robust vascular ingrowth in an ex vivo chorioallantoic membrane (CAM) assay, driven by the synergistic contributions of its hydrophilic, bioactive matrix chemistry and micro/macroporous topology.

Prior oxygen‐releasing scaffolds, such as PCL/CaO_2_ composites, have demonstrated that supplemental oxygen can enhance cell survival and promote robust bone repair in critical‐sized defects;^[^ ^5^, ^20^, ^93^, ^94^ ^]^ however, their hydrophobic matrices and slow degradation kinetics can limit early cell–matrix interactions, vascularization, and homogeneous oxygen distribution within the defect. COSnPPOD addresses these limitations by integrating a hydrophilic GelMA‐PEGDA matrix for rapid cell attachment, with nanoshell‐encapsulated CaO_2_ to temper peroxide hydrolysis for controlled oxygen release. The Primitive‐type TPMS architecture further promotes uniform mass transport, deep tissue infiltration, and spatially homogeneous neotissue formation.^[^ ^39^, ^42^ ^]^ This synergy between controlled oxygenation and curvature‐driven topological cues likely underpins the enhanced osteogenesis and neovascularization observed in vivo.

Mineralization observed along the inner surfaces of the COSnPPOD scaffolds suggests that the combination of topological guidance and bioactive oxygen support accelerated integration and bone‐like matrix formation. Partial scaffold persistence at week 8 with localized calcification indicates ongoing remodeling and potential for scaffold‐mediated tissue replacement. No gross evidence of fibrotic encapsulation, necrosis, or chronic immune response was noted in or around the implants.

In summary, the COSnPPOD scaffold enhanced bone regeneration in vivo without triggering systemic or local toxicity. The combination of controlled oxygen delivery with curvature‐driven topological design presents a promising strategy for addressing clinical challenges associated with large or poorly vascularized bone defects. Additionally, although CaO_2_ chemistry can generate H_2_O_2_, bulk ROS measurements in thick hydrogels have limited interpretability without species‐ and space‐time resolution and targeted rescue controls, given the hormetic signal versus distress behavior of ROS in osteogenesis. Our study therefore emphasizes pH equivalence, cell function readouts, and in vivo concordance, with future work incorporating Catalase and N‐acetylcysteine (NAC) as controls and species‐specific reporters to dissect redox contributions. Furthermore, future studies will explore the kinetics of neovascularization, scaffold resorption, and cell recruitment using lineage tracing, vascular perfusion imaging, and immune profiling to further elucidate COSnPPOD's mechanisms of action.

## 3 Conclusion

COSnPPOD, a VL‐DLP 3D printed Primitive‐TPMS hydrogel integrating CaO_2_–Si core–shell NPs in a GelMA‐PEGDA matrix, addresses early hypoxia while providing a tissue‐permissive architecture. The scaffold showed an early, tapering oxygen‐release kinetic compatible with the avascular window of bone tissue repair; maintained cytocompatibility in vitro with preservation of canonical osteogenic factors and marked Spp1 upregulation, and significantly enhanced bone regeneration in a murine calvarial defect model without local or systemic toxicity. These outcomes support a mechanism in which short‐term oxygenation synergizes with curvature‐driven, interconnected porosity to promote osteogenesis and vascularization. We acknowledge limitations of this study included the voxel‐limited sensitivity in µCT‐based microporosity analysis, and our emphasis on functional readouts (i.e., pH, in vitro cell behavior and responses, and in vivo repair) rather than species‐resolved redox assays. To address these limitations, future work will quantify in situ O_2_ and NP loading with standardized assays, incorporate catalase or *N*‐acetylcysteine rescue and redox biosensors, and assess vascular perfusion/maturity and cell recruitment (pericyte/endothelial markers, lineage tracing). Collectively, these data position COSnPPOD as a translatable, oxygen‐releasing scaffold strategy for poorly vascularized or large bone defects.

## 4 Experimental Section

### CaO_2_–Silica NP Synthesis

Synthesis of the CaO_2_–Silica NPs followed a modified protocol adapted from Wu et. al.^[^ ^50^ ^]^ To produce the hollow silica NPs a nanoemulsion, oil‐in‐water (O/W) process was used, which involved the following chemicals and reagents: cetyltrimethylammonium bromide (CTAB, Sigma) as the cationic surfactant, tetraethyl orthosilicate (TEOS, Sigma) as the silica source, and CaO_2_ (Calcium Peroxide, Sigma) as the therapeutic loaded within the NPs. A formulation of diH_2_O/CTAB/TEOS/CaO_2_ with a molar ratio of 224 000/1/132/44.9 was utilized for this process. Two solutions were prepared for the reaction—a water‐phase solution which consisted of CTAB (9 mg) dissolved in diH_2_O (100 mL), prepared in a 250 mL round‐bottom flask, and an oil‐phase solution which consisted of olive oil (400 µL, cooking grade), TEOS (730 µL), and CaO_2_ (80 mg), prepared in a 20 mL flat bottom vial. Both reaction solutions were kept under continuous stirring conditions, 1000 rpm for oil‐phase and 500 rpm for oil‐phase. The reaction was executed by pipetting 100 µL of the oil‐phase solution dropwise into the water‐phase solution, generating droplets of ≈6 µL, every 10 min until the oil‐phase solution was exhausted, to create the O/W nanoemulsion. Next, the reaction mixture was sonicated in an ultrasonic bath for 2 h at room temperature and then left in capped tubes to sit at room temperature, undisturbed, for 24 h. Upon completion of the reaction, the NPs were isolated from the water‐phase by filtration with a ceramic Buchner funnel using medium‐filtration filter paper. The NPs were then washed multiple rounds in sequence with ethanol and diH_2_O to remove unreacted chemicals remaining after the completion of the reaction and filtered a final time using a ceramic Buchner funnel overnight. Finally, the NPs were dried in an oven at 60 °C for 1 week or until completely dry, at which point they were ready for combination with the 3D printing ink.

The theoretical upper limits for the loading capacity (LC) and encapsulation efficiency (EE) of the NPs were calculated based on the feed stoichiometry of the synthesizing reaction. Specifically, given the canonical sol–gel mass yield of silicon dioxide (SiO_2_) from TEOS of 0.288, the SiO_2_ component of the total mass of NPs obtained from 730 mL TEOS is 0.730 mL × 0.933 g mL^−1^ × 0.288 = 0.196 g. With 0.08 g of CaO_2_ fed, LCmax = (80 mg/(80 mg + 196 mg)) × 100 = 29 wt%. Furthermore, from this estimation EEmax = 100% based on the definition of the bound. However, this is a conservative upper bound; measured LC and EE will be lower due to loss of encapsulated CaO_2_ during washing and filtration.

### Ink Preparation and 3D Printing

3D printing ink for hydrogel printing was composed of GelMA and PEGDA as the polymeric components, lithium phenyl‐2,4,6‐trimethylbenzoylphosphinate (LAP, Fisher Scientific) as the photoinitiator (PI), tartrazine (Fisher Scientific) as the photoabsorber (PA) and Dulbecco's phosphate buffered saline (DPBS, Gibco/Sigma/Corning) as the base. GelMA was synthesized based on a previously developed protocol^[^ ^37^ ^]^ to achieve a medium‐high degree of methacrylation (MD > 85%).^[^ ^95^, ^96^, ^97^, ^98^ ^]^ The specific process consisted of the following steps. DPBS (100 mL) was warmed in a water bath maintained at 50 °C with continuous stirring at 250 rpm. Type A porcine skin gelatin (10 g, Fisher Scientific) was added to achieve a 10% w/v solution and allowed to fully dissolve overnight, maintaining 50 °C and stirring. After the complete dissolution of gelatin, Methacrylic Anhydrate (MA, 20 mL, Fisher Scientific) was added dropwise using a dropwise addition funnel at a rate of 0.5 mL min^−1^ to the reaction vessel. The reaction was terminated after 2 h using a 2 times dilution with DPBS. The mixture was then transferred to 12–14 kDa dialysis tubing and allowed to dialyze against diH_2_O for 1 week to remove salts and methacrylic acid, maintaining elevated temperature at 50 °C. Upon verification that dialysis is complete via observation that the dialysis bath maintains pH ≈ 7, GelMA was lyophilized for 1 week to obtain a porous white foam. GelMA foam was stored at ‐20 °C.

Control GelMA‐PEGDA 3D printing ink was prepared by first warming 10 mL of DPBS to 40 °C under shaking at 300 rpm and adding LAP (0.05 g) to achieve a 0.05% concentration, allowing the LAP to fully dissolve while maintaining temperature and shaking. Room temperature GelMA (1.0 g) was added to the DPBS once LAP was fully dissolved to obtain 10% GelMA concentration. GelMA was allowed to fully dissolve, at which point PEGDA (893 µL, Fisher Scientific) was added to achieve an equal concentration of 10%. The 10% w/w ratio for GelMA‐PEGDA ink formulation used in this study was based on the findings of the team's previous work showing rapid visible‐light crosslinking, tunable mechanical properties, and proangiogenic potential in ex vivo CAM model.^[^ ^37^ ^]^ Finally, Tartrazine (5.34 g) was added for a concentration of 1 mm. Once all components were fully dissolved and incorporated, the ink was ready for 3D printing. It was maintained at 37 °C and under shaking in preparation for printing.

CaO_2_–Si NP loaded treatment ink for COSnPPOD scaffolds was prepared by adding NPs at a concentration of 80 mg mL^−1^. The CaO_2_–Si NP‐laden GelMA‐PEGDA prepolymer ink formulation was kept temperature‐controlled to prevent premature gelation and was gently agitated until loading. To limit any sedimentation, on‐vat dwell time was minimized, printing commenced immediately after loading, and the ink was remixed between print jobs. Because the particles are submicrometer and the prepolymer is viscous, the Stokes settling displacement is negligible over the print duration (micrometers per hour), so the distribution remains effectively uniform during layer‐by‐layer fabrication.

3D printing was conducted using a visible light‐based DLP system (Gen 3 LUMEN X, CELLINK) with a PDMS coated vat. Printing parameters were kept constant for control and COSnPPOD scaffolds: projected blue light wavelength of 405 nm, light intensity of 32 mW cm^−2^, and layer exposure time of 10s per layer with first layer exposure increased to 40s for improved print bed adhesion. Layer thickness was varied for porous Primitive and disk constructs, at 50 and 100 µm, respectively.

The COSnPPOD scaffold's topological design was based on TPMS‐Primitive, as previously described by the team.^[^ ^37^, ^99^ ^]^ The TPMS geometry incorporated interconnected pores of 800 µm and wall thicknesses of 200 µm.^[^ ^37^ ^]^ For in vivo implantation studies, scaffolds were initially printed with a bounding volume of 4 mm diameter × 2 mm height; upon hydration, they expanded to ≈5 mm in diameter, enabling a precise press‐fit within the critical‐size mouse calvarial defect used in this study. For in vitro cell culture experiments, the same Primitive scaffolds were uniformly 2×‐scaled to 8 mm diameter × 4 mm height to facilitate ease of handling, improved cell study, and sufficient sample mass for imaging and molecular assays. All printed scaffolds were washed thoroughly in DPBS and stored at 4 °C until use. Printed scaffolds were washed and stored in DPBS until time of use.

### Measurement of Scaffold Physical Characteristics

Physical characteristics including swelling behavior and gel fraction of the 3D printed scaffolds were measured using 3D printed porous samples (*n* = 4 samples for each condition per test). Immediately following printing and cleaning the weight of all the specimens was measured and recorded as the initial weight (W0). The mass swelling ratio was then measured, per a previous protocol.^[^ ^98^ ^]^ To summarize, after incubation in DPBS at 37 °C for 24 h, samples were blotted with a KimWipe and their weight measured, denoted as the swollen weight (Ws). Next, samples were lyophilized and weighed again, recording the dry weight of the hydrogel scaffolds (Wd_24_). Following a standard calculation of Ws/Wd_24_, the mass swelling ratio was obtained. Next, to measure the gel fraction, initial lyophilized dry mass was recorded (Wd_0_), followed by sample incubation in DPBS at 37 °C for 72 h so as to fully remove any uncrosslinked polymeric residuals. Finally, the samples were again lyophilized, and the dry mass was recorded (Wd_72_). The gel fraction was then calculated as (Wd_72_/ Wd_0_).

### Assessment of Biodegradation and Resulting Changes in Mechanical Properties in an Enzymatic Environment

Two different scaffold geometries were utilized for the degradation study. The porous geometry was used to assess degradation weight loss, in alignment with the proposed treatment system, while the cylindrical disk geometry (diameter = 5 mm, height = 1 mm) was necessary to perform nanoindentation testing to assess the changes in mechanical properties of the hydrogel system in response to hydrogel degradation. Porous geometry could not be utilized for nanoindentation experiments due to their curvature and overall topology, which would bias the deflection values collected by the indentation probe. To perform the biodegradation study, first a solution of DPBS and Type II collagenase (Fisher Scientific) at a concentration of 20 µg mL^−1^ was prepared for the assessment. Cylindrical and porous scaffolds, *n* = 3 samples per geometry and group for a total of 12 samples, were 3D printed, placed into the collagenase solution and stored at 37 °C. Samples were removed, minimally dried, and weighed every 24 h for the first 7 days and every 7 days after the first week. The collagenase solution replaced at every weigh‐in and additionally twice more between each weigh‐in after the first week. % weight loss was calculated as

(1) $$\%\;Weight\;Loss=100\;\times \frac{W_{o}-W_{i}}{W_{o}}$$

where W*_o_* is the initial weight of the sample and W*_i_* is the weight of the sample at day *i*.

To assess the extent of degradation and the effect of the encapsulation of NPs in the COSnPPOD scaffold, nanoindentation testing was performed using the optical fiber‐based interferometry nanoindenter (Pavone, Optics11Life) on the disk hydrogel samples at day 0 and day 14 of the biodegradation study. A nanoindentation probe with a tip radius of 26 µm and a stiffness of 0.46 N m^−1^ was utilized for the micromechanical testing. Indentations were performed as peak load single “pokes” with a max load of 1 µN. Data postprocessing was performed using the DataViewer software (Optics11Life). Young's Modulus and Effective Young's Modulus were calculated from the load‐indentation curve of each performed indentation, with a minimum of 20 indents per sample included in the analysis. Indents were excluded a priori, if the trace showed nonmonotonic loading/unloading, poor model fit (*R* ^2^ < 0.95), spurious contact detection (e.g., adhesion artifact), or clear probe slip/edge or pore contact evidenced by discontinuities. These objective criteria were applied uniformly across groups.

### Assessment of the Release Rate of the Therapeutic Agent from the COSnPPOD Scaffold

The gradual release of the CaO_2_–Si NPs from the COSnPPOD scaffold was verified by immersing porous COSnPPOD scaffolds into DPBS at 37 °C. Scaffolds were removed from the DPBS and placed into fresh DPBS every 24 h for 1 week and the optical density (OD) of the supernatant was measured via Ultraviolet‐Visible (UV–Vis) Turbidimetry on a plate reader with an absorbance wavelength of 300 nm.

The optimal absorbance wavelength for the CaO_2_–Si NPs was determined experimentally. First, OD values of CaO_2_–Si NP dilutions (80 and 40 mg mL^−1^, *n* = 6 per group) in DPBS and blank DPBS across a range of spectra (250, 300, 320, 350, and 380 nm) were recorded. 300 nm was chosen because it maximized the signal‐to‐blank (ΔOD) while remaining in a region where the solvent had negligible absorbance and the plate/cuvette remained transmissive. Moreover, linearity of the data was verified at this wavelength over the working range and obtained an *R* ^2^ value of 74% with stable OD readings, confirming sufficient linearity for the purposes of this study. This is consistent with turbidimetric practice:^[^ ^100^ ^]^ select a UV–vis wavelength where the continuous scattering tail of submicrometer particles gives strong, monotonic concentration dependence and minimal matrix interference.

Release of the NPs was quantified as a normalized release amount using the following relationship

(2) $$Normalized\;Release=\frac{OD_{i}-OD_{\mathrm{PBS}}}{OD_{0}-OD_{\mathrm{PBS}}}\;$$

where OD_0_ is the absorbance at day 0, OD*_i_* is the absorbance at day *i*, and OD_DPBS_ is the absorbance of a control well of fresh DPBS.

In addition, the pH level over the course of 7 days cell‐free culture was monitored. Porous control and COSnPPOD scaffolds (*n* = 3 per group) were incubated in α‐MEM (10% FBS, 1% pen/strep) at 37 °C, 5% CO_2_ without medium changes for 7 days (2 mL per well; 12‐well plates). To capture early transients, pH was recorded every 2 h over the first 6 h, and once daily thereafter through day 7 using Orion Lab Star PH211 (Thermo) with automatic temperature compensation. For each well and time point, three consecutive readings were averaged. This cell‐free assay complements the normalized NP‐release profile by reporting early hydrolysis/redox transients in the absence of cell metabolism while avoiding boundary‐condition biases inherent to in‐scaffold dissolved‐oxygen probes.

### Microstructure and Compositional Analysis of CaO_2_–Silica NPs and COSnPPOD Scaffolds

CaO_2_–Si NPs were dispersed in deionized water, gently sonicated for 5 min, and drop‐cast onto aluminum stubs with carbon tape. After air drying in a dust‐free container, samples were sputter coated with Au/Pd. Then, samples were imaged by field‐emission SEM at an accelerating voltage of 5 kV using the secondary electron detector. Representative fields were acquired to assess particle size distribution, sphericity, and surface texture.

For ultrastructure, similarly, NPs were dispersed in DI water, sonicated, and drop‐cast onto carbon‐coated copper TEM grids; excess liquid was wicked, and grids were air‐dried. Imaging was performed on a JEOL NEOARM operated in STEM mode at 200 kV (point resolution 0.0783 nm). Bright‐field and HAADF‐STEM (high‐angle annular dark‐field STEM) micrographs were acquired at 30 000x and 200 000x to visualize particle morphology and core–shell structure. Elemental analysis used the integrated EDX silicon‐drift detector under identical beam conditions; spectrum‐imaging (SI) maps for Si and Ca were collected and overlaid on HAADF images to resolve elemental distributions across individual particles. EDX spectra were background‐subtracted and quantified using the vendor's standardless routine.

Scaffold microstructure was examined by SEM at multiple magnifications with an accelerating voltage of 5 kV. EDX mapping focused on Si, O, and Ca to assess the distribution of the inorganic phase within the hydrogel matrix. As noted in Section 2, localized deformation observed in scaffolds reflects lyophilization/orientation artifacts rather than loss of structural integrity. Additionally, 3D printed scaffolds were embedded in optimal cutting temperature compound (OCT) following serial DPBS washes, cryosectioned on a Cryotomy Cryostar NX70, and stained using the FAST protocol of Leung et al.^[^ ^56^ ^]^ Stains included tartrazine and Alcian Blue/Safranin O to visualize matrix features and provide contrast for NP‐laden regions. Sections were imaged under identical acquisition settings across groups using bright‐field inverted microscope.

Internal architecture and porosity were assessed by µCT following PTA contrast staining, following a previously established published protocol.^[^ ^101^ ^]^ Briefly, a 0.3% w/v PTA solution was prepared in DI water. Scaffolds were first washed overnight in DPBS in 4 °C, then transferred into 3 mL of PTA solution and stained at room temperature for 24 h on an orbital shaker (150 rpm). After staining, each scaffold was washed in DI water (30 mL) for 45 min (nine 5‐min washes, fresh DI water each wash). For scanning, the stained scaffold was placed in a 1.8 mL microcentrifuge tube completely filled with Di water, wrapped with KimWipes to prevent motion. µCT scans were acquired on a SkyScan 1276 system (Bruker microCT, Kontich, Belgium) with the following parameters: 60 kV source voltage, 167 µA current, 0.5 mm Al filter, 5 µm pixel size resolution. Reconstructions were carried out in NRecon (Bruker). Raw image stacks (16‐bit TIFF) were imported into Amira‐Avizo Software (Thermo Fisher Scientific) for all postprocessing, 3D reconstruction/visualization, segmentation, and porosity analysis, following the vendor's porosity workflows.

### Protein Adsorption Study

VL‐DLP 3D printed scaffolds were sterilized prior to in vitro experiments through a protocol utilizing both ethyl alcohol and UV sterilization. First, the scaffolds were transferred into 70% ethyl alcohol and sterilized in a biosafety hood under UV for 30 min. Scaffolds were then rinsed with sterile DPBS to remove the remaining ethanol. Next, samples were laid out to sterilize under UV for 2 h per side. The scaffolds were lightly wetted with DPBS and rewetted every 30 min to prevent dehydration.

Sterilized and pre‐wetted 3D printed porous geometry scaffolds were placed in MC3T3 culture medium (composed of α‐MEM (Gibco), 10% FBS (Thermo Fisher), and P/S (100 µg mL^−1^, Thermo Fisher) and incubated at 37 °C overnight. After incubation the scaffolds were transferred and washed 3 times with DPBS in a 24‐well plate to remove any nonadhered proteins. Adhered proteins were removed from the hydrogel scaffolds through a 2‐step Sodium Dodecyl Sulfate (SDS, Fisher Scientific) removal process. 1% SDS solution (500 µL) was added to each well and the scaffolds were crushed thoroughly using a metal spatula. The mixture was left to incubate for 2 h at room temperature on a shaker and the SDS was collected at the end of the incubation period. SDS (500 µL) was again added to each well, incubation was repeated overnight, and the final round of SDS was collected. The final protein concentration in the collected SDS solution was quantified using the Micro‐BCA protein assay kit (Thermo Fisher).

### Cytocompatibility Assessments

The 3D printed hydrogel COSnPPOD scaffolds were evaluated for cytocompatibility using a preosteoblast MC3T3‐E1 cell line. MC3T3 cells were cultured in α‐MEM supplemented with FBS and P/S at the same concentrations as the protein adsorption study. In vitro studies were conducted in two parts: cylindrical disk control and COSnPPOD hydrogel samples (diameter = 10 mm, height = 1 mm, *n* = 3 samples per assay, per group) were seeded with a cell suspension (20 µL) containing 5×10^4^ cells to assess the 2D cell viability, spreading, and proliferation; MC3T3 cells were seeded onto a 12 well plate with a cell suspension (20 µL) containing 5×10^4^ cells and cultured with a 0.4 µm pore size transwell insert (Fisher Scientific) which contained porous (at 2x scale) hydrogel control and COSnPPOD scaffolds (*n* = 2 per assay, per group) to assess cell viability and proliferation in the presence of proposed treatment implant geometry.

Cell viability and spreading was assessed on cell‐seeded scaffolds on day 2 of the study, prior to osteogenic induction, using a Live/Dead assay kit (Thermo Fisher). Cell morphology and proliferation was assessed on day 2 using immunofluorescent staining for F‐actin/nuclei following fixation (4% PFA) and 3x DPBS washes. Specifically, it was stained with CellMask Actin Deep Red (Thermo) and Hoechst (Thermo) according to the manufacturer protocols. Fluorescence imaging was performed on the optical fiber‐based interferometry nanoindenter equipped with inverted fluorescent microscopy (Pavone, Optics11Life) and image postprocessing was conducted using Fiji, an ImageJ software package.

### In Vitro Assessment of Osteogenic Differentiation

Transwell cultured porous hydrogel scaffold group alone was used to assess osteogenesis in vitro. MC3T3 cells were cultured in standard cell culture medium for 48 h prior to replacing the cell culture medium with osteogenic medium, composed of the previously prepared MC3T3 media and supplemented with 50 µg mL^−1^ of ascorbic acid and 10 mm β‐glycerophosphate. Cells were trypsinized from the transwell after 3 and 8 days of culture after substitution of osteogenic medium. CyQuant Cell Proliferation assay kit (Thermo Fisher) was used to quantify cell proliferation.

Gene expression analysis was performed on day 8 of the in vitro transwell study according to the manufacturer's protocols. RNA was extracted from collected cells using TRIzol (Invitrogen) followed by purification with the GeneJET RNA Purification Kit (Thermo Fisher Scientific). The concentration of RNA was measured using a NanoDrop Microvolume Spectrophotometer (ThermoFisher Scientific) and samples were normalized to the lowest total RNA concentration. Next, cDNA was synthesized from the purified RNA after genomic DNA (gDNA) digestion, using SuperScript IV VILO Master Mix with ezDNase (Invitrogen). Finally, RT‐qPCR was performed using PowerTrack SYBR Green Master Mix (Applied Biosystems) with the following primers for the genes of choice: Alp1, Bglap. Runx2, Col1a, and Spp1, forward and reverse primer sequences listed in Table S1 (Supporting Information). The RT‐qPCR reactions were conducted on a Quantstudio 6 Pro system (Applied Biosystems). Gapdh and L32 were tested as the housekeeping gene, and the final gene expression results were normalized to L32 using the Comparative Ct (2−ΔΔCt) method.

### Mouse Calvaria In Vivo Study

Animal surgical work in this study was performed under the protocol reviewed and approved by Institutional Animal Care and Use Committee (IACUC; Protocol No. A00005664) at Mayo Clinic, USA. Primitive porous hydrogel scaffolds were 3D printed under sterile conditions according to the standard protocol. After creating a centered, critical‐sized calvarial defect (5 mm diameter) in the albino mice (Figure S3, Supporting Information), the COSnPPOD hydrogel treatment scaffold was placed into the opening. As a positive control, a GelMA‐PEGDA porous scaffold without CaO_2_–Si NPs was inserted into the defect site. Additionally, as a negative control, empty defects were left without a scaffold. The defect skin was then instantly closed using 4‐0 nonresorbable Vicryl sutures. 4 replicates were prepared for each group. 8 weeks after implantation, the mice were sacrificed, and the cranial tissues were cut off, along with select soft tissue organs, and fixed in a 10% paraformaldehyde (PFA) solution.

µ‐CT scanning of the cranial tissues was performed at the UTCT laboratory on the Zeiss Xradia 620 Versa, Germany. After scanning, 3D reconstruction of the bone tissues was generated using Avizo Amira software and new bone area was calculated through a quantitative analysis. After decalcifying using an ethylenediaminetetraacetic acid (EDTA) decalcifying solution containing hydrochloric acid from Thermo Fisher Scientific, the calvarial defect tissues were mounted and cryosectioned similar to the hydrogel samples. The sectioned samples were then stained with H&E, and Masson Trichrome stained according to manufacturer protocols. Stained sections were imaged using a Brightfield inverted microscope. Quantitative analysis of the area fractions of collagen in Masson Trichrome stained sections was performed using Fiji, an ImageJ software package.

### Immunofluorescent Staining for OPN and VEGF

Remaining cryo‐sectioned slices from the in vivo samples were used for immunofluorescent staining (*n* ≥ 3 group). Tissue areas were outlined with a hydrophobic PAP pen and rehydrated in DPBS. Sections were permeabilized and blocked in 5% bovine serum albumin (BSA) in DPBS at 37 °C for 1 h in a dark, humidified chamber. Primary antibodies against osteopontin (OPN; 1:200 dilution, Proteintech) and vascular endothelial growth factor (VEGF; 1:200 dilution, Thermo Fisher) were diluted in antibody solution (5% BSA, 0.5% Triton X‐100 in DPBS) and applied to sections for overnight incubation at 4 °C. The following day, slides were rinsed briefly in DPBS and washed three times for 10 min each in PBST (DPBS + 0.1% Tween‐20) at room temperature with gentle orbital shaking. Secondary antibodies (1:1000 dilution in antibody solution) were applied for 2 h at room temperature, followed by DAPI nuclear counterstaining (2 µg mL^−1^ in DPBS) for 10 min. Slides were washed as above, with a final wash in DPBS, and mounted with antifade mounting medium. Coverslips were allowed to harden overnight before storage at 4 °C (short‐term) or ‐20 °C (long‐term). Imaging was performed using identical acquisition parameters across all groups to enable direct comparison of fluorescence intensity and spatial distribution.

### Statistical Analysis

Prism 10 software was utilized for all performed statistical analyses. All data were presented as mean ± standard deviation, with *n* ≥ 3 independent experimental replicates per group. Analysis was conducted using Welch's *t*‐test, one‐way ordinary or Brown–Forsythe and Welch analysis of variance (ANOVA), or two‐way ANOVA followed by Fisher's LSD test for multiple comparisons, as appropriate based on the type of data. Statistical significance was denoted as: *: *p* < 0.05, **: *p* < 0.01, ***: *p* < 0.001, ****: *p* < 0.0001.

Total of *n* = 4 mice (biological replicates) per group was used in vivo. A post hoc sensitivity analysis (Methods‐S1, Supporting Information) for the histology endpoint (one animal‐level mean per mouse) showed a large ANOVA effect size (*η^2^* = 0.739; Cohen's *f* ≈ 1.68). With *k* = 3 groups, *n* = 4 per group, and *α* = 0.05, this corresponds to > 80% power to detect between‐group differences of this magnitude. For pairwise contrasts on histology, animal‐level Hedges’ *g* values exceeded the minimal detectable effect for *n* = 4 per group (two‐sided, *α* = 0.05), indicating adequate sensitivity to the observed effects.

## Conflict of Interest

Dr. Elder has consulting agreements with SI Bone and Depuy Synthes, receives clinical trial support from SI Bone and Stryker, is on the medical advisory board and has stock options in Injectsense, and serves on the executive board of the International Society for HyÂ­drocephalus and CSF Disorders. Other authors declare that they have no known competing financial interests or personal relationships that could have appeared to influence the work reported in this paper.

## Author Contributions

A.B.T. and M.T. conceived and designed the study. A.B.T. led the experiments and analyses. A.B.T., A.G., C.K., D.C., and M.T. performed experiments, conducted literature review, and analyzed and interpreted the data. M.A.P., B.D.E., and M.T. performed the in vivo experiments. V.A. performed the µCT experiments. A.B.T. wrote the original manuscript draft. M.T. supervised the study and provided primary resources. S.S., R.G., B.D.E., and J.E.B. provided supporting resources. All authors reviewed and revised the manuscript and approved the final version.

## Supporting information



Supporting Information

## Data Availability

The data that support the findings of this study are available from the corresponding author upon reasonable request.
