# Supplementary material for: Visible Light Induced DLP‐Printed Oxygen‐Releasing TPMS Scaffolds Mitigate Early Hypoxia in Bone Defects
Source: Adv Healthc Mater. 2025 Sep 23;15(3):e02735. doi: 10.1002/adhm.202502735 (PMC12817115; doi:10.1002/adhm.202502735)
Supplement: Supplementary file 1 — Supporting Information [file ADHM-15-0-s001.docx]

**Visible Light Induced DLP-Printed Oxygen-Releasing TPMS Scaffolds**

**Mitigate Early Hypoxia in Bone Defects**

Anastasia B. Timoshenko^1^, Ali Ghasemkhani^1^, Chanul Kim^2^, Domenic J. Cordova^1^, Maria Astudillo Potes^3^, Valeria Aceves^4^, Indranath Mitra^5^, Justin E. Bird^6^, Ryan Gray^7^, Stephanie Seidlits^2^, Benjamin D. Elder^3,8^, Maryam Tilton^1^*

^1^ Walker Department of Mechanical Engineering, The University of Texas at Austin, USA

^2^ Department of Biomedical Engineering, The University of Texas at Austin, USA

^3^ Department of Orthopedic Surgery, Mayo Clinic, Rochester, USA

^4^ College of Natural Sciences, The University of Texas at Austin, USA

^5^ Department of Applied Science, William & Mary, USA

^6^ Department of Orthopedic Oncology, The University of Texas MD Anderson Cancer Center, USA

^7^ Department of Nutritional Sciences, Dell Pediatric Research Institute, The University of Texas at Austin, USA

^8^ Department of Neurologic Surgery, Mayo Clinic, Rochester, USA

*Corresponding author: [maryam.tilton@austin.utexas.edu](mailto:maryam.tilton@austin.utexas.edu)

# Supporting Information


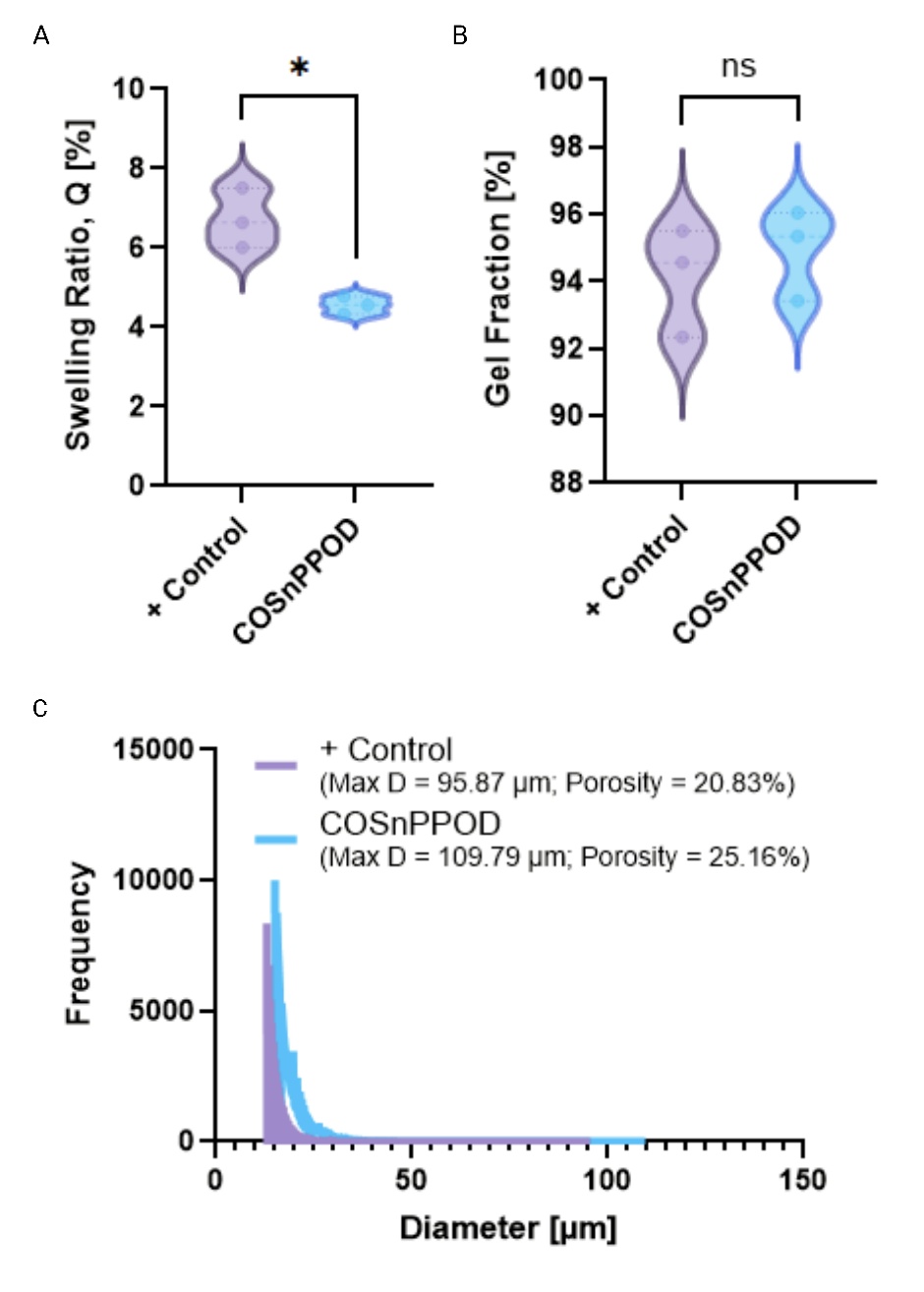


**Figure S1: Supplemental data for structural analysis of the hydrogel scaffolds.** (A) Swelling Ratio of the COSnPPOD and GelMA-PEGDA TPMS porous scaffolds, displaying a statistically larger degree of swelling in the positive control hydrogels that do not contain CaO_2_ NPs. (B) Gel Fraction of the COSnPPOD and positive control porous scaffolds; COSnPPOD scaffolds showed a marginally larger gel fraction value compared to positive control. (C) Pore Diameter vs. Frequency of appearance along with maximum pore diameter and average porosity values for COSnPPOD and positive control hydrogels as measured from Micro-CT imaging of the samples. Quantitative results are presented as mean ± SD. Statistical significance relative to positive control was calculated using Welch’s t-test; *p* < 0.05 is designated as statistically significant. Statistical significance: *: *p* < 0.05, **: *p* < 0.01, ***: *p* < 0.001, ****: *p* < 0.0001.


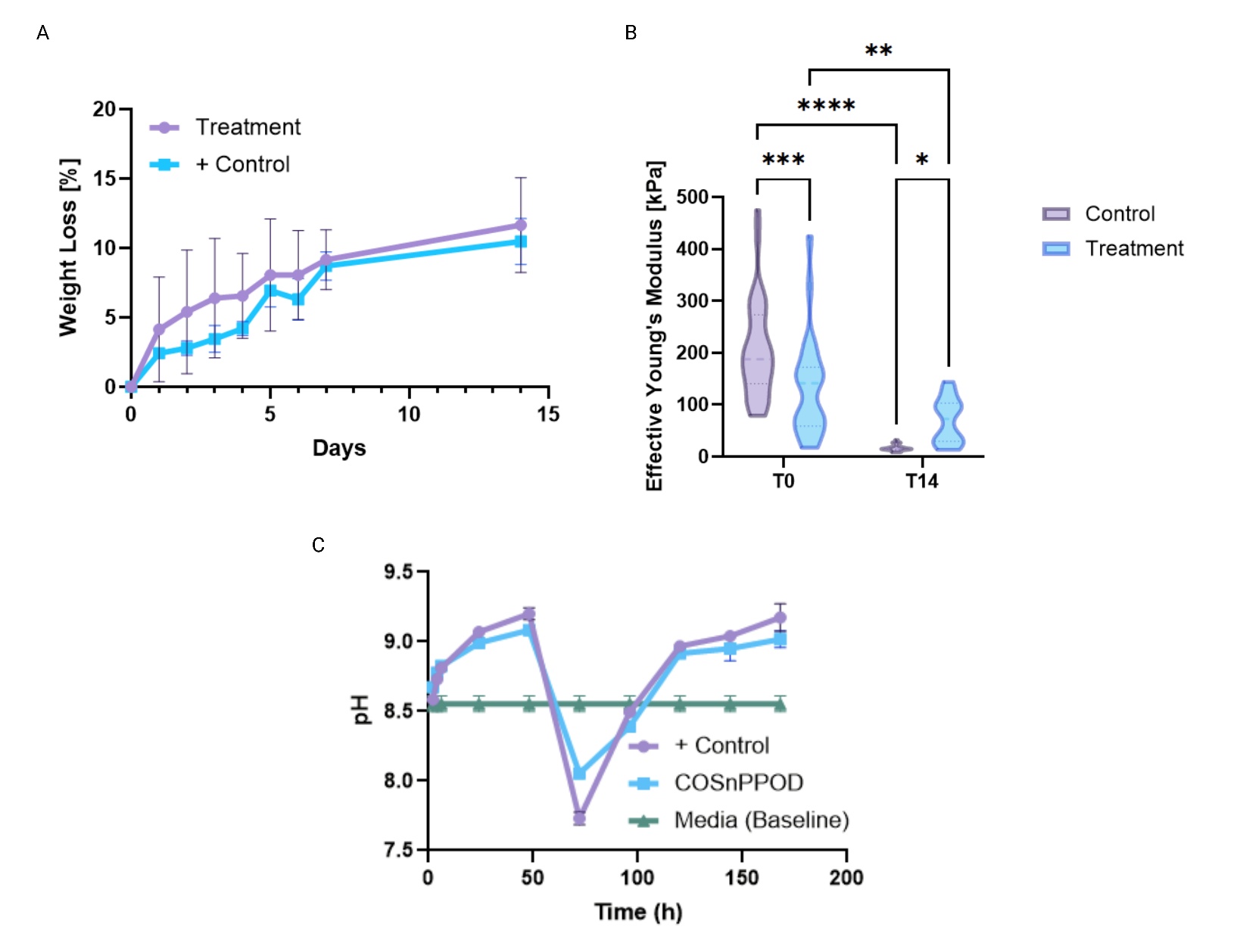


**Figure S2: Supplemental data for in vitro biophysical characterization.** (A) % Weight Loss of the COSnPPOD and control disk-shape scaffolds, which were used in the nanoindentation experiment, over the course of the biodegradation study. (B) Compiled Effective Young’s Modulus data for the positive control and COSnPPOD disk scaffolds over the course of the biodegradation study. (C) pH variation of the culture media (without cells) for GelMA-PEGDA and COSnPPOD scaffolds compared to baseline media pH over the course of 7 days of culture. Quantitative results are presented as mean ± SD. Statistical significance relative to positive control was calculated using two-way ANOVA and Fisher’s LSD test for multiple comparisons; *p* < 0.05 is designated as statistically significant. Statistical significance: *: *p* < 0.05, **: *p* < 0.01, ***: *p* < 0.001, ****: *p* < 0.0001.

**Table S1: Primer sequences used for RT-qPCR experiments.**

| **Gene** | **Forward Primer Sequence** | **Reverse Primer Sequence** |
| --- | --- | --- |
| Bglap | CCTGAGTCTGACAAAGCCTTCA | GCCGGAGTCTGTTCACTACCTT |
| Runx2 | GGCACAGACAGAAGCTTGATGA | GAATGCGCCCTAAATCACTGA |
| Alpl | CACAGATTCCCAAAGCACCT | GGGATGGAGGAGAGAAGGTC |
| Col1a1 | GCTTCACCTACAGCACCCTTGT | TGACTGTCTTGCCCCAAGTTC |
| Spp1 | CCCGGTGAAAGTGACTGATTCT | GATCTGGGTGCAGGCTGTAAA |
| Gapdh | ACTCAAGATTGTCAGCAAT | CCATCCACAGTCTTCTGGGT |
| L32 | CCATCTGTTTTACGGCATCATG | TGAACTTCTTGGTCCTCTTTTTGA |


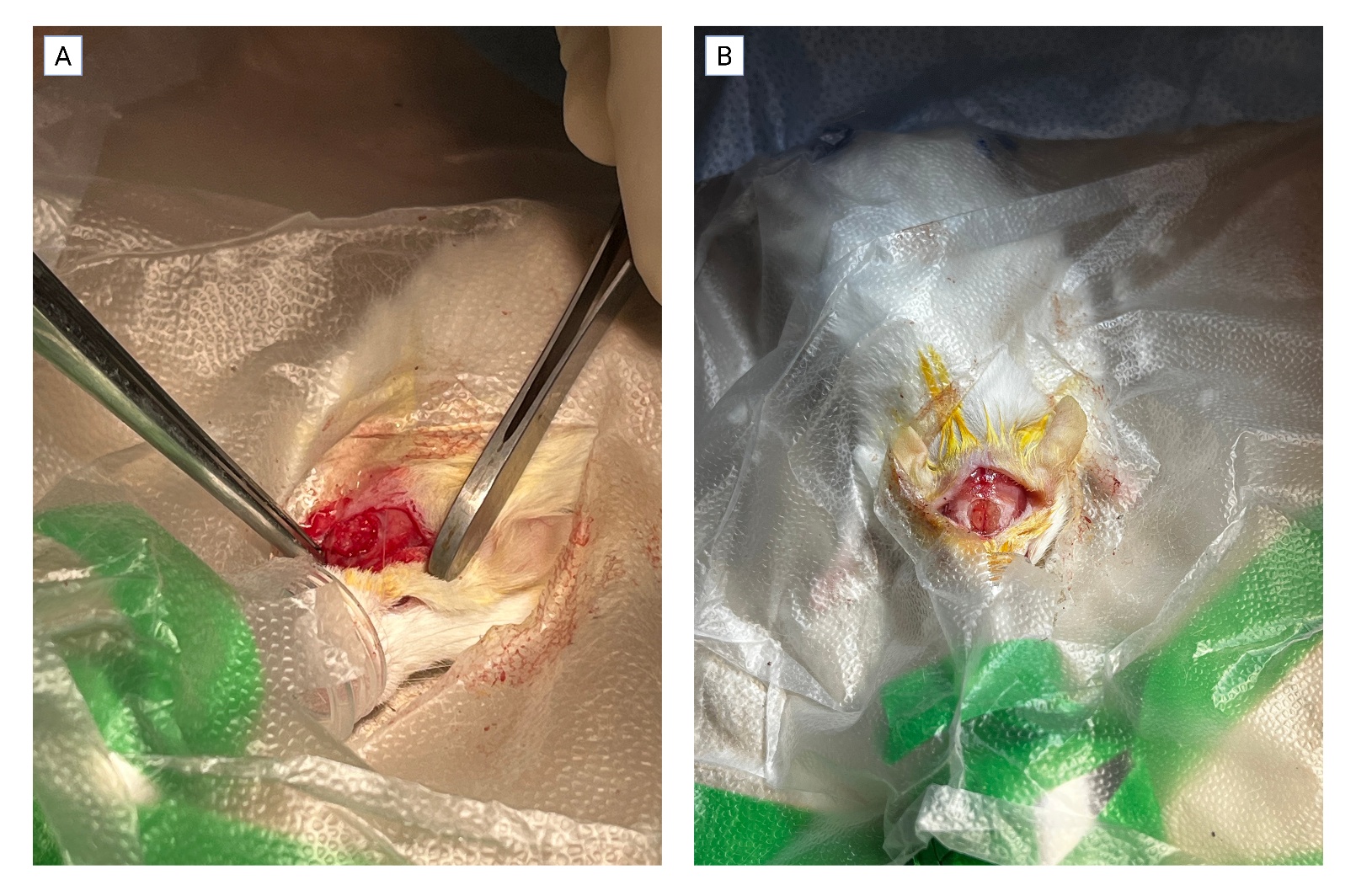


**Figure S3: In process images of the in vivo animal surgical procedure.** (A) Opening of the incision to expose the mouse calvaria. (B) Example of the 5 mm cranial defect created in albino mice specimens.

**Methods-S1: Power Analysis for Determination of In Vivo Sample Size.**

Endpoint: Collagen area fraction (%) from Masson’s trichrome, computed as the mean of ≥ 5 fields per animal and analyzed at the animal level (*n* = 4 per group; *k* = 3; α = 0.05). Group means ± SD: -Ctrl 18.55 ± 18.57, +Ctrl 41.37 ± 13.66, Treatment 68.91 ± 8.30. From this data, it was calculated that *η^2^* = 0.739 and the following equation was used to calculate Cohen’s *f*:

$$Cohen^{'}s f= \sqrt{\frac{\eta^{2}}{(1-\eta^{2})}}$$

This equation yielded a Cohen’s *f* = 1.68. Under these parameters, power exceeds 80% for detecting effects of this magnitude. Pairwise Hedges’ *g* (animal level) further quantified contrasts: Trt vs +Ctrl, *g* ≈ 2.12; Trt vs -Ctrl, *g* ≈ 3.05; +Ctrl vs -Ctrl, *g* ≈ 1.22.

Minimal detectable effect (MDE): For two-group comparisons with *n* = 4 per group, *α* = 0.05 (two-sided), the MDE is approximately *d* ≈ 2.37 for 80% power. The animal-level *g* values for Treatment vs controls exceed this threshold on the histology endpoint, consistent with adequate sensitivity.
